# Mycobacteria trehalose dimycolate interactions with host Mincle remodel blood-brain barrier junctions for brain invasion

**DOI:** 10.1016/j.celrep.2025.116661

**Published:** 2025-12-11

**Authors:** Megan I. Hayes, Sumedha Ravishankar, Jonathan K. Shanahan, Adam J. Fountain, Lalita Ramakrishnan, Cressida A. Madigan

**Affiliations:** 1 School of Biological Sciences, University of California, San Diego, La Jolla, CA 92037, USA; 2 Molecular Immunity Unit, Cambridge Institute of Therapeutic Immunology and Infectious Diseases, Department of Medicine, University of Cambridge, Cambridge CB2 0QH, UK; 3 Medical Research Council Laboratory of Molecular Biology, Cambridge CB2 0QH, UK; 4 These authors contributed equally; 5 Lead contact

## Abstract

Tuberculous meningitis is unique among bacterial meningitides because it occurs in two temporally separated steps: mycobacteria first invade the brain, then form infected macrophage aggregates called Rich foci, which later erode the meninges. Here, using transparent zebrafish larvae, we detail the first step—brain invasion. We find that whereas elsewhere in the body mycobacteria disseminate within phagocytes, only extracellular mycobacteria reach the brain microvasculature. There, they adhere to the microvascular endothelium and grow into microcolonies. These microcolonies induce endothelial tight junction reorganization, creating transient gaps through which bacteria enter the brain and infect microglia to initiate Rich foci. This reorganization is induced by mycobacterial surface glycolipid trehalose dimycolate interacting with its receptor, Mincle. Strikingly, the pathogens *Mycobacterium tuberculosis* and *Mycobacterium marinum* and the saprophyte *Mycobacterium smegmatis* can all invade the brain via this pathway. Thus, *M*. *tuberculosis* initiates meningitis, the deadliest form of tuberculosis, using an ancestral determinant important for environmental fitness.

## In brief

Using a zebrafish model of brain infection, Hayes et al. find that mycobacteria infect the brain not within macrophages, but as extracellular bacteria that attach to endothelial cells of the blood-brain barrier. Using a glycolipid, mycobacteria trigger the opening of gaps in endothelial cell junctions, allowing transit into the brain.

## Graphical Abstract

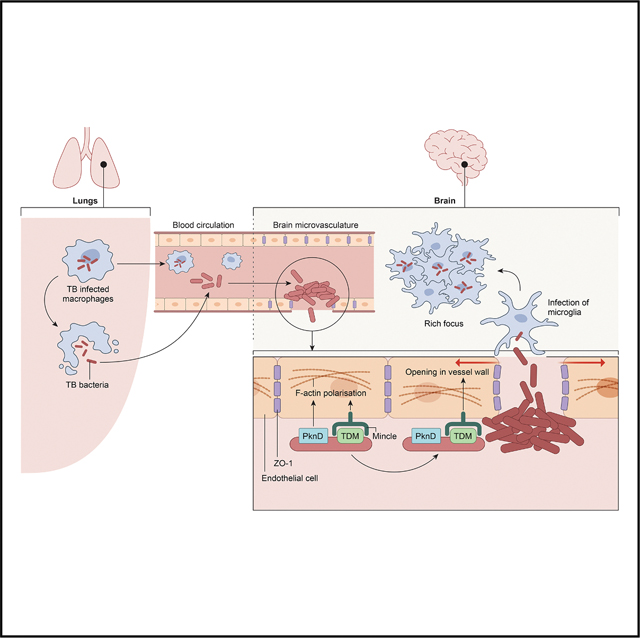

## INTRODUCTION

Meningitis, one of the most serious bacterial diseases, carries a high mortality despite antimicrobial treatment.^1,2^ Tuberculous meningitis is no exception; almost uniformly fatal without antitubercular chemotherapy, mortality remains 20%–40% with treatment, increasing to >80% for drug-resistant tuberculosis (TB).^3–5^ Survivors often suffer lifelong neurological deficits, and children are disproportionately affected, compounding the burden placed by this devastating disease.^3,4^

Meningitis occurs when organisms enter the circulation and invade the meninges through the endothelial cells of blood-brain barrier (BBB) vessels.^1,2,6^ To protect the brain from toxins and pathogens, the BBB downregulates transcytosis and increases expression of specialized tight junction proteins that deter traversal between cells.^7,8^ Therefore, few bacteria regularly cause meningitis.^2^ Most are extracellular commensals that can breach mucosal barriers, enter the circulation, and traverse the BBB to cause acute meningeal infection.^1^ In human brain microvascular endothelial cells (HBMECs), these bacteria are inferred to cross the BBB by transcytosis or lysis.^2^ *Neisseria meningitidis* is exceptional in that it breaches endothelial cell tight junctions to traverse paracellularly.^2,9,10^ While epidemiological studies suggest that *M*. *tuberculosis* also invades the brain through the circulation,^4,11–13^ meningitis does not occur directly upon meningeal seeding.^1,11,12,14^ Histopathological analyses of brains from fatal tuberculous meningitis cases suggest a distinct pathophysiology with disease occurring in two stages. First, *M*. *tuberculosis* invades the brain or meninges and forms an immune aggregate or granuloma called the Rich focus. Months later, meningitis occurs as the granuloma matures and becomes necrotic.^11,12,14,15^

Identifying the mechanism by which mycobacteria invade the brain has been stymied by the lack of animal models, where these steps can be visualized.^4,16^ The zebrafish larva’s optical transparency and genetic amenability enable real-time delineation of the earliest events of mycobacterial pathogenesis.^17–21^ It has also been used to examine *M*. *marinum*’s traversal of the BBB and the blood-retinal barrier^22,23^ and group B *Streptococcus’s* and *Cryptococcus neoformans’s* traversal of the BBB.^24–26^

We performed live imaging studies in zebrafish larvae to experimentally corroborate human autopsy studies showing that mycobacterial entry from blood vessels into the brain produces the Rich focus. We then dissected the entry process and found that extracellular bacteria first attach to brain microvascular endothelial cells using the serine threonine kinase PknD, reported to promote *M*. *tuberculosis*-mediated actin polymerization and invasion of HBMECs.^27,28^ However, our detailed *in vivo* analyses revealed that PknD’s role in brain invasion is limited to promoting attachment to the endothelium. We went on to discover that the attached bacteria then invade the brain by an unexpected mechanism of paracellular transit: transient tight junction remodeling, mediated by their surface glycolipid trehalose 6,6′-dimycolate (TDM). Our findings explain the peculiarities of tuberculous meningitis, particularly that it occurs in two steps, with the intervening period between them being subclinical.

## RESULTS

### Circulating extracellular mycobacteria invade the brain to establish Rich foci

**Figure 1.**
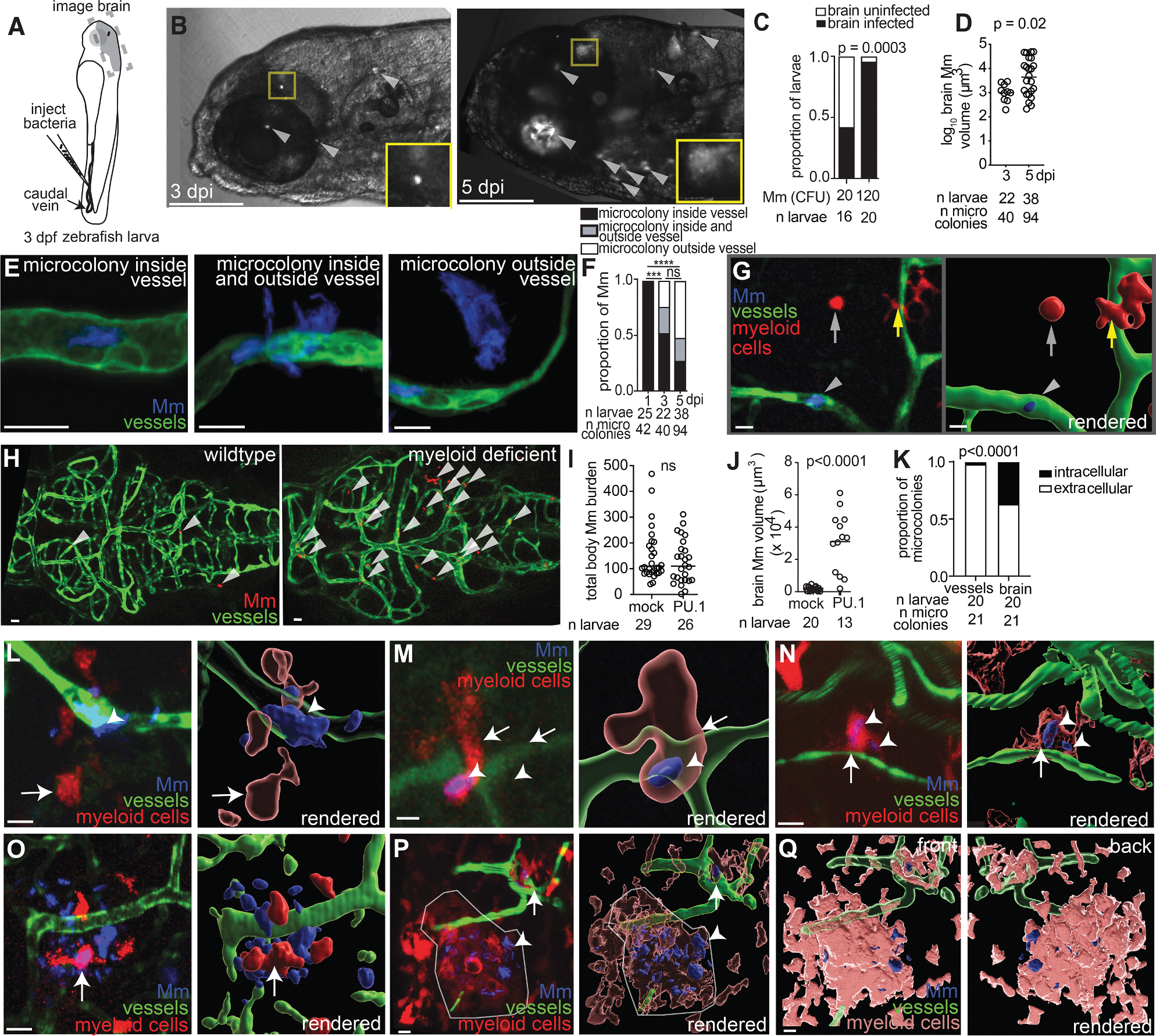
Extracellular *M. marinum* invades the brain and then infects microglia to form nascent Rich foci (A) Schematic drawing of a 3 dpf zebrafish larva showing the caudal vein infection site (injection needle) and the brain region where imaging was performed (dashed box). (B) Image of brain region of the same larva at 3 dpi (left) and 5 dpi (right), infected with ~120 CFU fluorescent *M. marinum* (Mm) showing microcolonies (arrowheads). Boxes indicate a microcolony that has enlarged between 3 and 5 dpi. Scale bars, 100 μm. (C) Proportion of larvae with brain infection after inoculation with ~20 or ~120 CFU Mm. Horizontal bars, means; s; Fisher’s exact test. Representative of 3 independent experiments. (D) Volume of fluorescent Mm microcolonies in the brain at 3 and 5 dpi in larvae infected with ~120 CFU. Horizontal bars, means; Student’s *t* test. Representative of 3 independent experiments. (E) Representative confocal images of 3 dpi larvae with green-fluorescent blood vessels infected with blue-fluorescent Mm showing microcolonies inside, partially outside, and completely outside brain blood vessels. Scale bars, 10 μm. (F) Quantification of images from experiment in (E). Fisher’s exact test, ***p < 0.001, ****p < 0.0001, and ns, not significant. Representative of 3 independent experiments. (G) Representative confocal image of a 3 dpi transgenic larva with green-fluorescent blood vessels and red-fluorescent myeloid cells (arrows), including microglia (yellow arrow), infected with blue-fluorescent Mm (arrowhead). The right panel shows a 3D rendered version of the image in the left panel. Scale bars, 10 μm. (H) Representative images of green-fluorescent brain microvasculature in 2 dpi wild-type and myeloid-deficient (PU.1 morphant) larvae infected with red-fluorescent Mm (arrowheads). Scale bars, 10 μm. (I) Mm burden per larva quantified by fluorescent pixel counts from experiment in (H). Horizontal bars, means; ns, not significant; Student’s *t* test. Representative of 3 independent experiments. (J) Mm volume in the brains of larvae in (I). Horizontal bars, means; Student’s *t* test. Representative of 3 independent experiments. (K) Proportion of Mm microcolonies in the brain blood vessels or brain tissue of 3 dpi larvae infected with Mm; Fisher’s exact test. Representative of 3 independent experiments. (L–O) Representative images of interactions between blue-fluorescent Mm (arrowheads), green-fluorescent blood vessels, and red-fluorescent myeloid cells (arrows) in a 3 dpi larva infected with Mm. The right panels show the 3D rendered images of the left panels. Scale bars, 10 μm. Extracellular Mm crossing a vessel with microglia close by (L). Crossing Mm is being taken up by a myeloid cell (M and N). In the larva from (B), Mm that have already crossed completely are associated with aggregating myeloid cells (O). (P and Q) A 4 dpi larva with a red-fluorescent granuloma (Rich focus) (circled) forming near point of exit of blue-fluorescent Mm from green-fluorescent blood vessel. 3D rendering of Rich focus with transparent myeloid cells (right) to show infected (arrowhead) and uninfected cells (arrow). Scale bars, 10 μm (P). 3D rendering of the front (left) and back (right) of a Rich focus with opaque myeloid cells to show extracellular Mm. Scale bars, 10 μm (Q).

To confirm that *M. marinum* invades the brain of zebrafish larvae via a hematogenous route, we intravenously injected ~20 or ~120 colony-forming units (CFUs) into larvae at 3 days post-fertilization (dpf) and imaged their brains serially some days later (Figure 1A). Most observations were made at 3 days post-infection (dpi) (6 dpf), when the zebrafish BBB has matured by suppressing transcytosis and increasing tight junction integrity.^29^ Discrete foci of living bacteria (or microcolonies) appeared in the brain by 3 dpi (Figure 1B, left panel). The frequency of brain dissemination correlated with inoculum: inocula of 20 and 120 bacteria produced brain dissemination in 42% and 96% of animals, respectively (Figure 1C). After a single intravenous injection, new microcolonies comprising approximately 8–12 bacteria appeared over time and expanded by 5 dpi (Figure 1B, compare left and right insets, 3 and 5 dpi, respectively). Thus, both continual dissemination from the blood and *in situ* growth contributed to increased total bacterial volume in the brain (Figure 1D).

To determine if the bacteria had invaded the brain tissue from the microvasculature, transgenic larvae with green-fluorescent vascular endothelial cells (*kdrl:GFP*)^30^ were infected with blue-fluorescent *M*. *marinum* and imaged sequentially over several days. At 1 dpi, *M*. *marinum* microcolonies were exclusively within brain blood vessels and began traversing the vessel wall by 3 dpi, before entering the brain at 5 dpi (Figures 1E and 1F). Together, these experiments confirmed that circulating *M*. *marinum* invades the brain.

As *M*. *tuberculosis* resides predominantly in myeloid cells that can disseminate it throughout the body, it has been assumed that mycobacteria enter the brain within myeloid cells.^2,4,31^ However, mice lacking myeloid cells still develop mycobacterial meningitis,^32^ and extracellular *M*. *tuberculosis* can invade and cross HBMECs,^28^ although a predominant role for myeloid cells, which are absent in this *in vitro* system, could not be evaluated. In zebrafish larvae, as in humans, myeloid cells play a major role in *M*. *marinum* tissue dissemination from the circulation into distal body sites.^20,33,34^ One study reported that *M*. *marinum* used myeloid cells to traverse the BBB, and only when phagocytes were depleted were free bacteria reported to traverse via presumed transcytosis.^22^

To test if phagocytes are involved in mycobacterial brain invasion, we infected *mpeg1:dsRed;kdrl:GFP* larvae^35^ that have red-fluorescent myeloid cells and green-fluorescent blood vessels. In contrast to the prior study,^22^ few *M*. *marinum* microcolonies in the brain microvasculature were within the myeloid cells in any of the five animals examined at 4 dpi (Figure 1G). Myeloid cells were observed only outside the blood vessels and within the brain tissue,^33^ many displaying the ramified morphology typical of microglia, brain-resident macrophages (Figure 1G, yellow arrow). No myeloid cells were observed within the brain blood vessels of uninfected animals either; in three animals examined, all 111 *mpeg1*-positive myeloid cells observed were extravascular microglia. This is consistent with mammals that restrict cellular traffic into the brain, likely to reduce the risk of dysregulated inflammation in this vulnerable organ.^7,36^ To further confirm that myeloid cells were dispensable, we depleted animals of myeloid cells by morpholino knockdown of the myeloid transcription factor *spi1b* (also called *pu.1*).^34^ At 2 dpi, when the overall bacterial burdens throughout the larvae in wild-type and *pu.1* morphant animals were equivalent, there were significantly more microcolonies in the *pu.1* morphant brains (Figures 1H–1J). Thus, these data suggest that myeloid cells *deter* rather than promote mycobacterial brain invasion, likely because monocytes are restricted from the brain vasculature, thereby keeping mycobacteria out of the brain.

Our observation that brain dissemination occurs predominantly via extracellular mycobacteria might seem at odds with findings that infecting bacteria are rapidly taken up by macrophages and reside within them in the early stages of infection.^34,37^ However, dead macrophages can release extracellular mycobacteria into circulation that could enter the brain blood vessels.^37^ If so, mycobacteria invading the brain would have been macrophage-resident before becoming extracellular. To test this, we used *Mi marinum* expressing a macrophage-activated promoter (*map49*) fused to GFP, in addition to constitutive red fluorescence (Figures S1A and S1B).^38^ Macrophage-resident mycobacteria continue to express GFP for some days after becoming extracellular, with the signal diluting due to replication. We found that the majority of brain microcolonies (61.5%) retained some GFP (Figures S1C and S1D), consistent with the release of extracellular mycobacteria by dead macrophages. This mechanism could reconcile our findings with the prior report surmising that brain infection is predominantly mediated by macrophages.^22^ As we and others observe, bacteria escaping from circulating monocytes may enter the brain extracellularly before infecting microglia.^27,32^ Therefore, our findings suggest that because myeloid cells are largely excluded from the brain vasculature even during systemic infection, extracellular mycobacteria released from macrophages are responsible for brain invasion.

### Mycobacteria infect microglia to initiate Rich foci

We next sought to confirm human autopsy studies suggesting that *M*. *tuberculosis* invasion of the brain or meninges initially results in the formation of granulomas termed Rich foci.^11,14,15^ Because *M*. *tuberculosis* can infect microglia in culture, it has been inferred that it infects these cells *in vivo* to initiate the Rich focus.^4,39^ Our finding that extracellular mycobacteria are responsible for brain invasion suggests that, after crossing the BBB, brain-resident microglia are infected first. At 3 dpi, several extracellular mycobacteria that had invaded the brain were within the microglia (Figure 1K). As mycobacteria traversed brain blood vessels, we observed microglia migrating toward the mycobacteria and phagocytosing them, irrespective of whether the bacteria were still partially within the blood vessel (Figure 1L) or had just entered the brain parenchyma (Figures 1M and 1N). Initially, the foci were composed entirely of microglia, resembling brain invasion by cryptococcus.^40^ Within days, infected and uninfected myeloid cells were recruited to form aggregates (Figures 1O–1Q and S2A–S2J), as expected from previous work. By 3 dpi, monocytes were recruited to aggregates, which we observed using intravenous injection of Hoechst, a nuclear dye that differentiates monocytes (Hoechst-positive) from microglia (Hoechst-negative), both of which were present in the aggregates^33^ (Figure S2K). Thus, extracellular mycobacteria invading the brain are rapidly phagocytosed by microglia, initiating brain granulomas resembling Rich foci.

### Mycobacterial attachment and growth on the microvascular endothelium are associated with F-actin rearrangements but not endothelial transcytosis or damage

**Figure 2.**
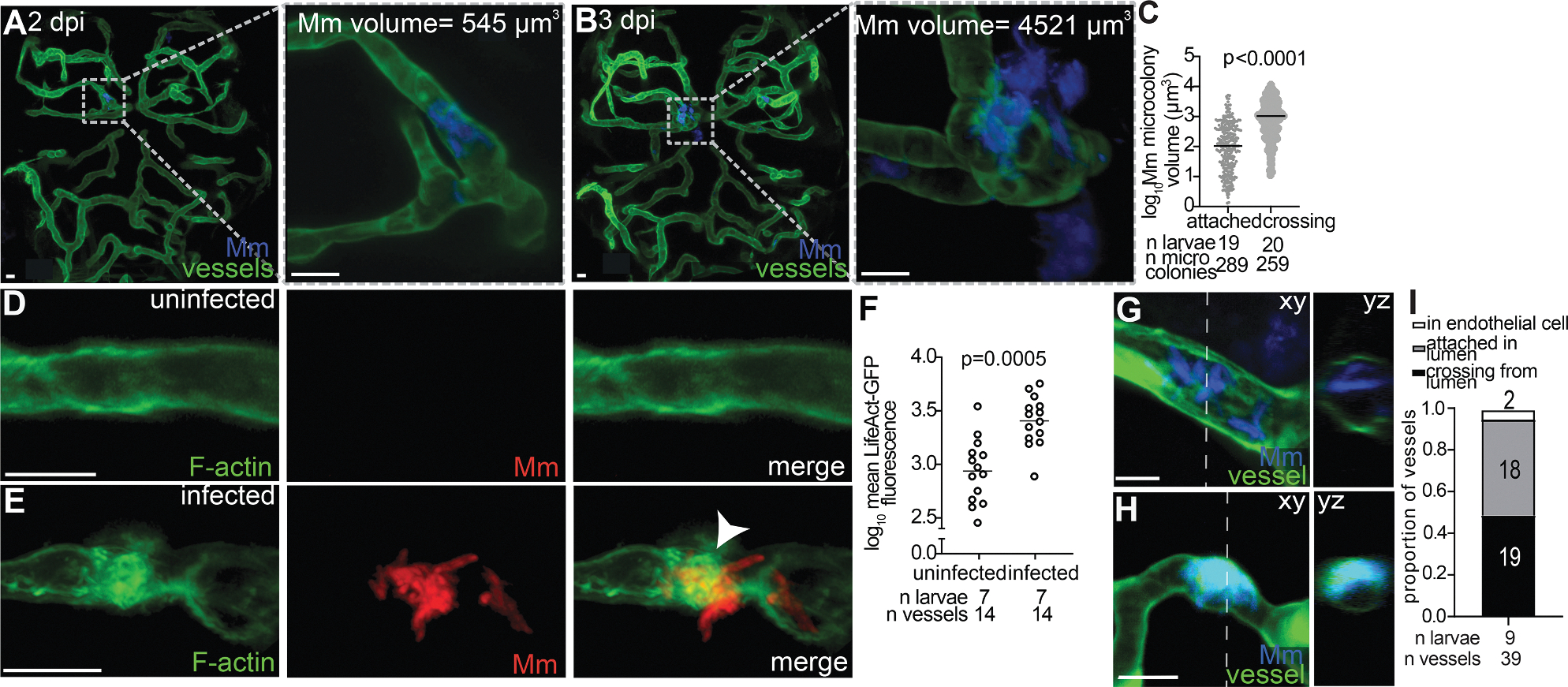
*M. marinum* microcolonies grow and recruit endothelial cell F-actin (A and B) Representative confocal images of the same larva brain with green-fluorescent blood vessels, infected with blue-fluorescent *M. marinum* (Mm). Insets highlight a microcolony inside the vessel at 2 dpi (A), crossing at 3 dpi (B). Scale bars, 10 μm. (C) Volume of attached and crossing Mm microcolonies at 1 dpi. Horizontal bars, means. Representative of 2 independent experiments. (D and E) Representative confocal images of uninfected (D) and infected (E) vessels from *flk:GAL4;UAS:LifeAct-GFP* larvae at 3 dpi with red-fluorescent Mm. Arrowhead, green-fluorescent F-actin accumulated around the Mm microcolony. Scale bars, 10 μm. (F) Quantification of F-actin in the infected vessel compared to the contralateral uninfected vessel from the same animal at 3 dpi. Horizontal bars, means; paired Student’s *t* test. Representative of 2 independent experiments. (G and H) Representative confocal images (*xy* axis) with optical cross sections (*yz* axis) of green-fluorescent vessels of 3 dpi larvae infected with blue-fluorescent Mm. Mm microcolony in blood vessel lumen, as indicated by the lack of co-localization of blue and green fluorescence. Scale bars, 10 μm (G). Mm inside the blood vessel endothelial cell, indicated by co-localization of blue and green fluorescence. Scale bars, 10 μm (H). (I) Proportion of vessels containing Mm in the lumen that are not crossing (gray), crossing (black), or inside of an endothelial cell (white) at 3 dpi. Representative of 4 independent experiments.

Having established that extracellular mycobacteria are responsible for brain invasion, we sought to understand the route that these bacteria use to cross the BBB. Using time-lapse confocal microscopy, we observed that circulating *M*. *marinum* occasionally became attached to the brain endothelium (Figure 2A). Serial monitoring of individual microcolonies suggested that mycobacteria cross the BBB where they initially attached (Figure 2B). Furthermore, the attached microcolonies grew *in situ* before crossing. For instance, the microcolony shown in Figures 2A and 2B had grown 8.3-fold between 2 dpi, when it was not yet crossing, and 3 dpi, when it was crossing. Overall, the microcolonies in the process of crossing were larger than those that were attached but not crossing (Figure 2C). Thus, to enter the brain, circulating mycobacteria adhere to endothelial cells, replicate *in situ* to form microcolonies, and cross the BBB at the attachment site. This could suggest that the *in situ* growth on the vessel wall facilitates brain invasion or that increased microcolony size facilitates crossing, irrespective of the *in situ* growth.

*M*. *tuberculosis* adherence to HBMECs was found to trigger actin rearrangements, leading the authors to infer that brain invasion occurs via transcytosis through the endothelial cells’ endocytic route.^27,28^ We confirmed that F-actin rearrangements occurred *in vivo*, using transgenic larvae expressing the GFP-tagged F-actin biosensor, LifeAct, in endothelial cells (*flk:GA-L4;UAS:Lifeact-GFP*).^41^ We observed that brain blood vessels had increased LifeAct-GFP where *M*. *marinum* contacted the endothelial cells, compared to blood vessels without bacteria (Figures 2D–2F). However, a few endothelial cells were infected (in 2/39 vessels where they had attached) (Figures 2G–2I), and none of the bacteria crossed the BBB (Figure 2H). Only attached bacteria that had not been internalized crossed the BBB (19 instances), suggesting that transcytosis is not a predominant mechanism of mycobacterial brain invasion (Figure 2I).

This apparent discrepancy with the HBMEC findings^27,28^ is readily explained by recent studies, showing that the downregulation of transcytosis does not occur *in vitro* but is integral to BBB function *in vivo*, including in larval zebrafish, and is mediated by pericyte interactions with endothelial cells, which would be absent in HBMECs.^6,29,42^

Having ruled out transcytosis, we wanted to determine whether mycobacteria invade the brain using lysis, similar to more common meningeal pathogens, such as *Streptococcus pneumoniae* and group B *Streptococcus*.^2,26,43,44^ Pathogenic mycobacteria have a membranolytic protein, ESAT-6, secreted by the ESX-1 secretion system, which can lyse cells in a contact-dependent manner and is required for full virulence in macrophages.^45,46^ A previous study found that BBB crossing in larvae with macrophages requires ESX-1, suggesting endothelial cell damage.^22^ In contrast, we found that in macrophage-depleted larvae, ESX-1 was dispensable for brain invasion (Figures S3A–S3E). Indeed, wild-type *M*. *marinum* did not result in endothelial cell staining by propidium iodide (PI), a cell-impermeant nuclear dye, in any of the 13 crossing events observed across 8 larvae, ruling out endothelial cell damage (Figure S3F). This is in stark contrast to group B *Streptococcus,* in which PI staining reveals extensive cell lysis.^44^ We cannot explain the differences between our ESX-1 findings and those reported previously, in that we did not find reduced ESX-1 mutant bacteria in the brain or damage to endothelial cells, which is the proposed mechanism by which ESX-1 promotes BBB crossing. Importantly, *M*. *tuberculosis* does not lyse HBMECs, consistent with our findings that mycobacteria do not lyse endothelial cells to cross the BBB.^28^ Given their barrier function, BBB endothelial cell membranes may be more resistant to bacterial cytolysins, making them able to resist ESAT-6 and other mycobacterial cytolysins, which might have weaker lysis activity than those of streptococci.

### Mycobacteria dynamically remodel endothelial cell tight junctions to create transient gaps through which they invade the brain paracellularly

**Figure 3.**
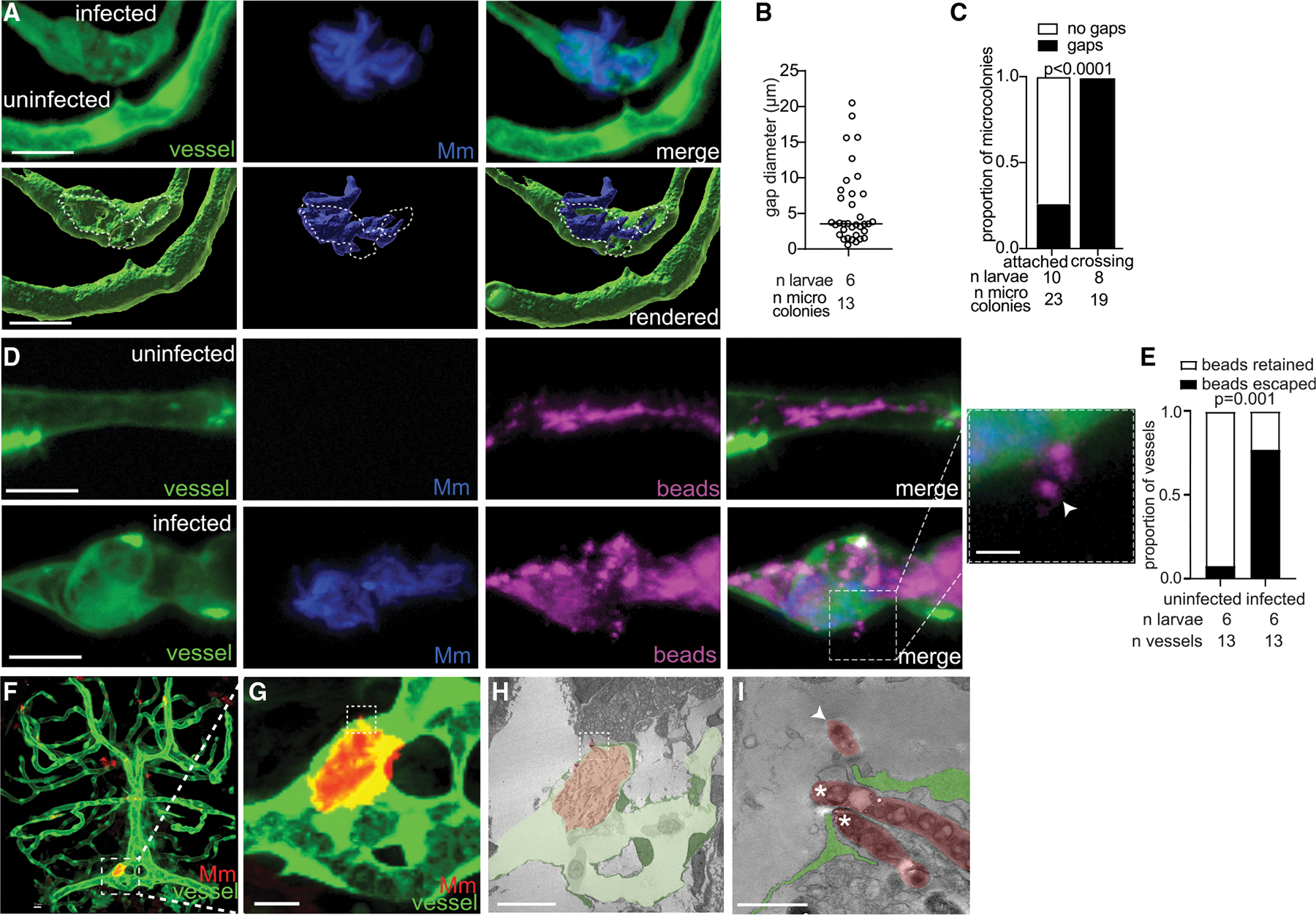
*M. marinum* crosses through permeable gaps in brain blood vessels (A) Representative confocal image (top) and 3D rendering (bottom) of an infected brain blood vessel near an uninfected vessel, from a 3 dpi larva with red-fluorescent blood vessels (pseudo-colored green) infected with blue-fluorescent *M. marinum* (Mm). The dashed circle indicates the border of the gap underneath the microcolony in the vessel. Scale bars, 10 μm. (B) Maximum diameter of vessel gaps formed by Mm microcolonies from larvae in (A). Horizontal bar, mean. Representative of 4 independent experiments. (C) Proportion of attached or crossing Mm microcolonies associated with blood vessel gaps. Fisher’s exact test. Representative of 2 independent experiments. (D) Representative confocal images of uninfected (top) or infected (bottom) green-fluorescent brain blood vessels from 3 dpi larvae infected with blue-fluorescent Mm and injected intravenously with far red-fluorescent 0.02 μm latex beads (pseudo-colored magenta) just prior to imaging. Scale bars, 10 μm. The inset shows beads leaking from the infected vessel (arrowhead). Scale bar, 5 μm. (E) Proportion of vessels with retained beads or those that escaped into the brain in uninfected and infected vessels from the experiment in (D). Fisher’s exact test. Representative of 2 independent experiments. (F and G) Brain confocal images of a 3 dpi larva with green-fluorescent vessels infected with red-fluorescent Mm, which were used to identify a crossing microcolony for transmission electron microscopy (TEM) in (H and I). The dotted box in (F), magnified in (G), indicates the area imaged by TEM. (H and I) TEM showing the microcolony (pseudo-colored red) primarily in the vessel lumen (pseudo-colored light green) with endothelial cells (pseudo-colored dark green). Scale bar, 10 μm (H). Two Mm bacilli (white asterisks) crossing between two endothelial cells (pseudo-colored green). Arrowhead, Mm that has fully crossed into the brain. Scale bar, 1 μm (I).

Having ruled out transcytosis and endothelial cell damage, we investigated whether mycobacteria might cross the BBB paracellularly, through endothelial cell junctions. Consistent with a paracellular route, we observed small openings—“gaps” (mean diameter 3.5 μm)—in the endothelial cell membrane where *M*. *marinum* was attached, but not in uninfected vessels (Figures 3A and 3B). Gaps were invariably associated with BBB crossing, as bacteria protruded through the gaps in the vessel (Figures 3A and 3C). We confirmed that these gaps allowed circulating materials to transit the BBB by imaging the brain immediately after injecting 0.02 μm fluorescent beads into the caudal vein of infected and uninfected animals. In infected animals, the beads escaped into the brain, whereas they remained within the vessels of uninfected animals (Figures 3D and 3E). Moreover, the beads entered the brain only in regions where microcolonies were attached (Figures 3D and 3E). Thus, microcolony attachment appears to trigger localized gaps in the microvascular endothelium through which mycobacteria invade the brain.

To investigate the presence of paracellular gaps in more detail, we used transmission electron microscopy (TEM). The TEM of the brain confirmed that most of a crossing microcolony was attached to endothelial cells of the vessel lumen (Figures 3F–3I), with two bacteria crossing the BBB (Figure 3I, white asterisks) through an otherwise intact cell junction (Figure 3I, green cells). This confirmed that mycobacteria enter the brain paracellularly between intact endothelial cells.

**Figure 4.**
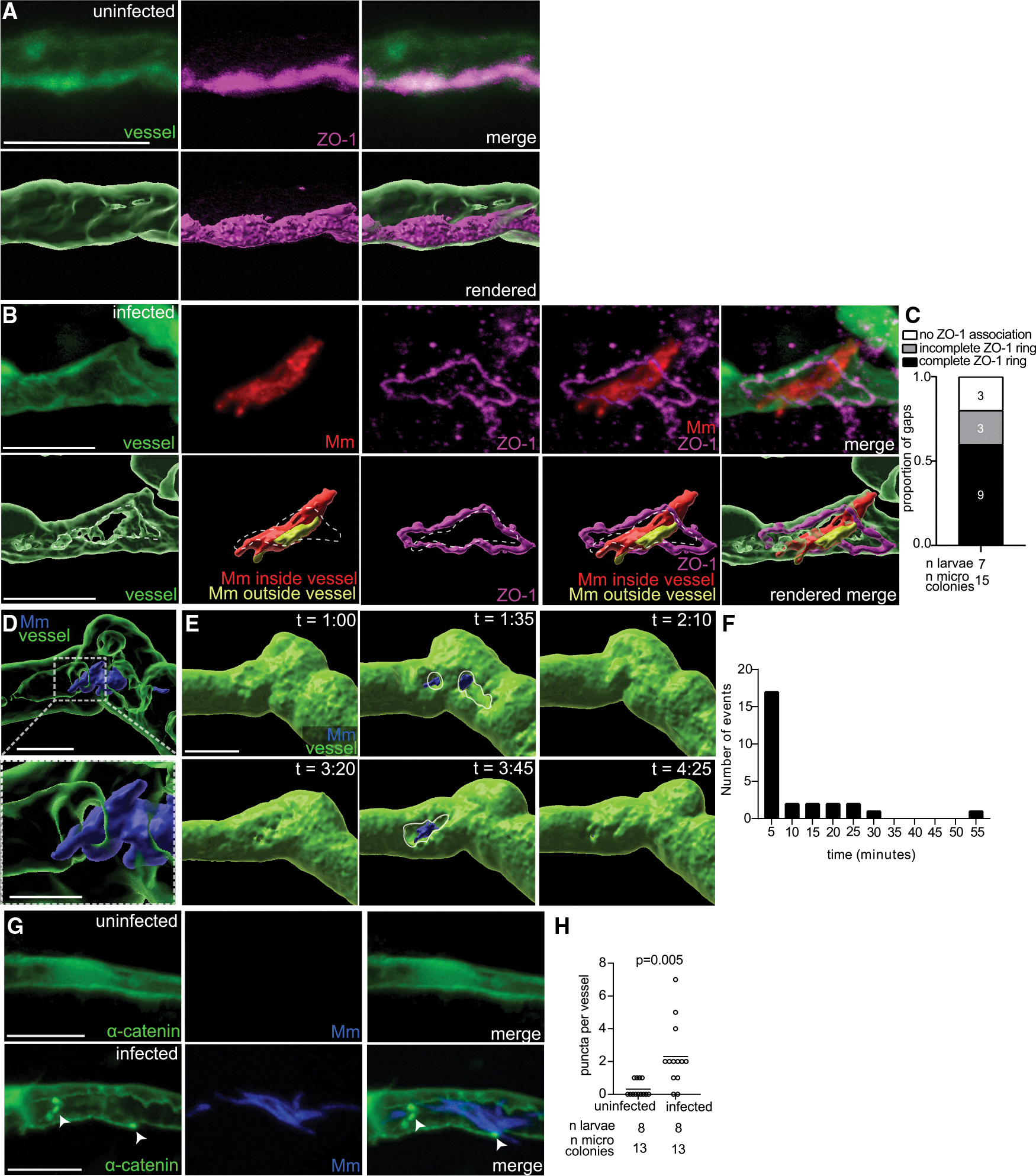
*M. marinum* reorganizes endothelial cell junctions to cross paracellularly (A and B) Representative confocal images of uninfected (A) and infected vessels (B) from 3 dpi larvae with green-fluorescent vessels infected with red-fluorescent *M. marinum* (Mm), fixed and stained with anti-ZO-1 antibody (pseudo-colored magenta). Bottom panels: 3D rendered versions of the top panels. In (B), 3D renderings show a gap (dashed circle), ringed by ZO-1 under Mm microcolony. Yellow indicates parts of the microcolony that have exited the vasculature and entered the brain. Scale bars, 10 μm. (C) Proportion of gaps associated with a complete, incomplete, or no ZO-1 ring for Mm microcolonies that are crossing blood vessels. Representative of 2 independent experiments. (D) 3D rendered images of red-fluorescent brain blood vessel (pseudo-colored green) in a 4 dpi larva infected with blue-fluorescent Mm, which is protruding from a gap, taken from Video S2. Scale bars, 10 μm. Boxed area is magnified in the bottom panel. Scale bars, 5 μm. (E) Sequential 3D rendered images from Video S2, showing gaps (outlined) forming and resealing near an Mm microcolony in a brain blood vessel. Time (t), h:min after the start of time-lapse video recording. Scale bars, 10 μm. (F) Histogram of frequency of duration of all gaps forming and closing in Video S2. The *x* axis details the 5-min time windows (e.g., 5: 0–5 min, 30: 26–30 min). (G) Representative confocal images of uninfected (top) and infected vessels (bottom) from a 3 dpi larva with green-fluorescent α-catenin, infected with blue-fluorescent Mm. Arrowheads indicate punctate α-catenin signal. Scale bars, 10 μm. (H) Total α-catenin puncta in uninfected and infected vessels. Horizontal bars, means; paired Student’s *t* test.

BBB function is maintained by multiple interconnected junctional complexes that promote adhesion of adjacent endothelial cells,^47^ including tight and adherens junctions.^7^ To cross the BBB paracellularly, these junctional complexes must undergo remodeling. We first investigated tight junctions, the most apical of these complexes,^47^ which also prevent pathogens from crossing the gut epithelium.^48^ To test if microcolony attachment alters tight junctions, we used an antibody to zonula occludens-1 (ZO-1), a cytosolic protein that regulates tight junctions.^7^ Vessels without microcolonies showed localization of ZO-1 to the cell border, creating a single clean seam defining the tight junction (Figure 4A). In contrast, ZO-1 was dramatically reorganized into ring-like structures that surrounded the gaps where mycobacteria protruded into the brain (Figures 4B and 4C; Video S1). We were struck by the presence of vessel gaps, given the importance of BBB integrity. Long-term time-lapse imaging resolved this apparent discrepancy, revealing that the gaps were transient, closing minutes after forming. During 7 h of video monitoring with images captured every 5 min, 27 gaps formed and closed (Figures 4D–4F; Video S2). The majority of the gaps (63%) closed within 5 min of forming and 33.3% within 30 min, with a single outlier (3.7%) remaining open for 55 min (Figure 4F). Open gaps allowed individual mycobacteria or microcolonies to protrude into the brain (Figures 4D and 4E; Video S2). Tight junction and adherens junction reorganization go hand in hand, owing to their close proximity and interconnectedness. To confirm that attached microcolonies reorganize adherens junctions, we imaged α-catenin, which stabilizes the junction and binds F-actin.^49^ Using larvae expressing α-catenin-GFP in endothelial cells *(flk1:α-catenin-eGFP)*,^41^ we found that vessels with microcolonies showed a 7.5-fold increase in GFP puncta, suggesting active reorganization of α-catenin (Figures 4G and 4H). Vessels without a microcolony showed a mostly uniform distribution of sparse α-catenin throughout the cell (Figure 4G). In sum, attached *M*. *marinum* microcolonies dynamically remodel both tight and adherens junctions, creating transient tight junction gaps in endothelial cells, through which some bacteria enter the brain.

### Mycobacterial PknD promotes brain invasion by promoting F-actin polymerization and attachment but does not cause tight junction remodeling

**Figure 5.**
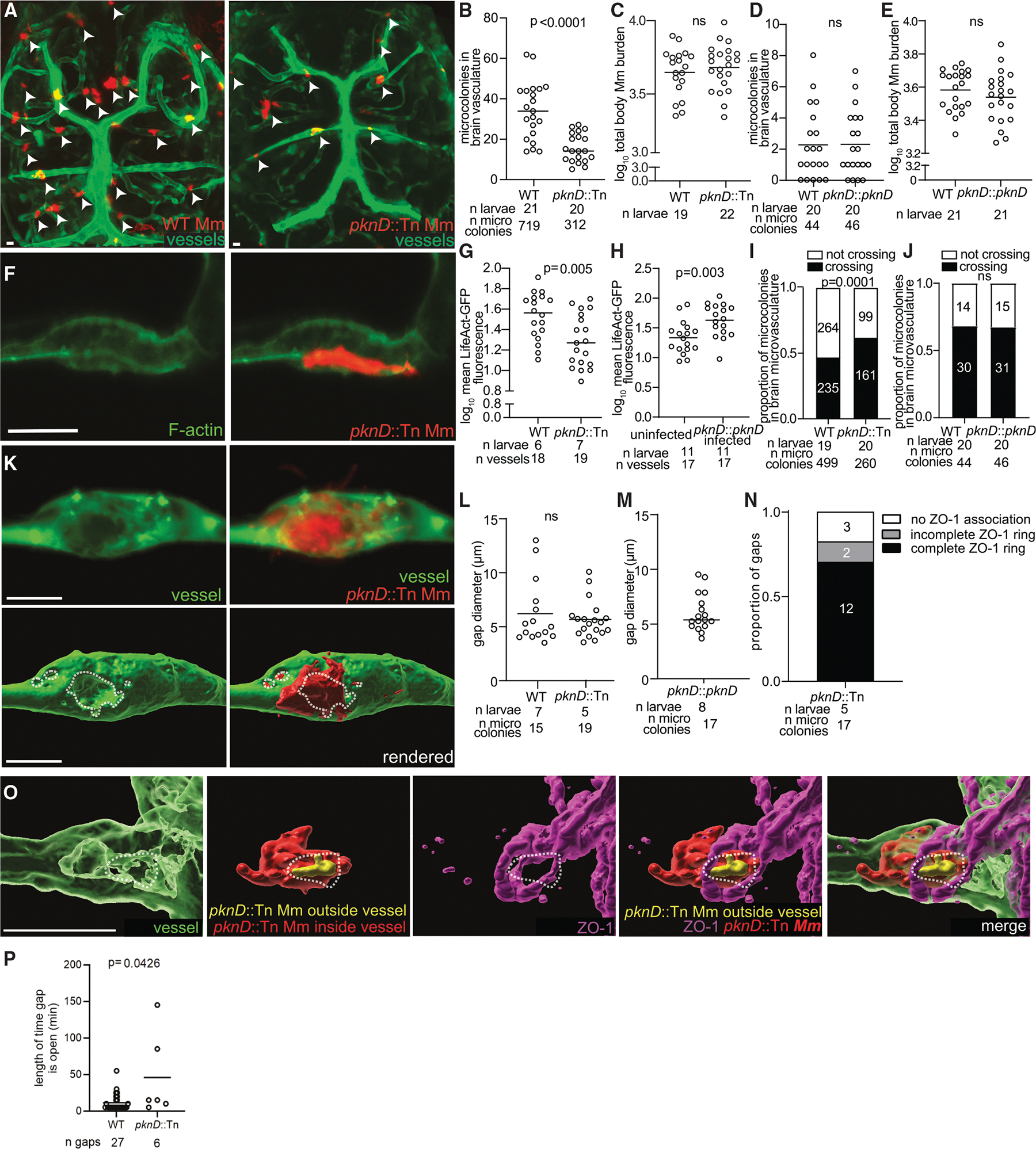
PknD contributes to *M. marinum* attachment, but not crossing through vessel gaps (A) Representative confocal images of the green-fluorescent brain vasculature in 1 dpi larvae infected with ~1000 CFU red-fluorescent wild-type (WT) *M. marinum* (Mm) (left), or ~3000 CFU red-fluorescent *pknD*::Tn Mm (right). Scale bars, 10 μm. (B) Total WT and *pknD*::Tn Mm microcolonies in the brain vasculature (attached and crossing). Horizontal bars, means; Student’s *t* test. (C) WT or *pknD*::Tn Mm burden per larva at 1 dpi quantified by fluorescent pixel counts (FPC) from the experiment in (A). Horizontal bars, means; Student’s *t* test; ns, not significant. (D) Total WT and *pknD*::Tn:*pknD* Mm microcolonies in the brain vasculature (attached and crossing). Horizontal bars, means; Student’s *t* test; ns, not significant. (E) WT and *pknD*::Tn:*pknD* Mm burden per larva at 1 dpi quantified by FPC. Horizontal bars, means; ns, not significant; Student’s *t* test. (F) Representative confocal images of red-fluorescent *pknD*::Tn Mm-infected vessels from *flk:GAL4;UAS:LifeAct-GFP* larvae at 1 dpi. Scale bar, 10 μm. (G and H) Quantification of F-actin in WT and *pknD*::Tn Mm infected vessels (G), or uninfected and *pknD*::Tn:*pknD* Mm. Horizontal bars, means; Student’s *t* test. Representative of 2 independent experiments. (I) Proportion of attached or crossing WT and *pknD*::Tn Mm microcolonies (excluding those in the brain) in blood vessels from larvae in (A); Fischer’s exact test. Representative of 2 independent experiments. (J) Proportion of attached or crossing WT and *pknD*::Tn:*pknD* Mm microcolonies (excluding microcolonies in the brain) in blood vessels. Fischer’s exact test; ns, not significant. (K) Representative confocal image (top) and 3D rendering (bottom) of a 2 dpi larva with green-fluorescent blood vessels infected with red-fluorescent *pknD*::Tn Mm. Dashed circle indicates the border of the gap underlying the microcolony in the vessel. Scale bar, 10 μm. (L and M) Maximum diameter of vessel gaps formed underneath Mm microcolonies infected with WT or *pknD*::Tn Mm (L), or *pknD*::Tn:*pknD* Mm (M). Horizontal bars, means; ns, not significant; Student’s *t* test. Representative of 2 independent experiments. (N) Proportion of gaps associated with a complete, incomplete, or no ZO-1 ring for *pknD*::Tn Mm microcolonies that are crossing blood vessels. (O) 3D rendered, representative confocal image from a 1 dpi larva with green-fluorescent vessels infected with red-fluorescent *pknD*::Tn Mm, then fixed and stained with anti-ZO-1 antibody (pseudo-colored magenta). Dashed circle, gap ringed by ZO-1 under *pknD*::Tn Mm microcolony. Yellow indicates parts of the microcolony that have exited the vasculature and entered the brain. Scale bars, 10 μm. (P) Length of time (min) that gaps remain open in the green-fluorescent vessels of a larva infected with WT or *pknD*::Tn Mm. Error bars, means; Student’s *t* test.

We next wanted to understand the mycobacterial factors that are important for endothelial attachment and tight junction remodeling. The *M*. *tuberculosis* serine/threonine kinase PknD was previously identified in a transposon mutant screen, which found that a PknD mutant had reduced dissemination to the brain in guinea pigs.^27^ In HBMECs, the *M*. *tuberculosis* PknD mutant displayed decreased invasion, and the authors inferred that transcytosis was occurring.^27^ Given our findings that *M*. *marinum* predominantly invades paracellularly, we hypothesized that, *in vivo*, PknD must promote F-actin rearrangements, which increase attachment to endothelial cells, tight junction remodeling, and gap formation. Compared to wild-type, we found fewer *M. marinum* PknD mutant bacteria attached to the brain microvasculature (Figures 5A–5C) and the absence of F-actin rearrangements (Figures 5F–5H), despite similar overall bacterial burdens. Complementing the PknD mutant with *M*. *marinum* PknD (Figures 5D and 5E, 5H, and 5J) rescued these defects, confirming that *M*. *marinum* PknD promotes brain invasion in zebrafish, similar to *M*. *tuberculosis* in guinea pigs.^27^

In addition to promoting attachment, if PknD-mediated F-actin rearrangements also caused cell junction remodeling to create gaps, then the PknD mutant should exhibit a crossing defect over and above its attachment defect. However, the PknD mutant, once attached, did not have a crossing defect. In fact, it crossed more often than the wild type (62% vs. 47%) (Figure 5I). Complemented PknD microcolonies crossed at a similar ratio to wild type, showing that this increased crossing was specifically caused by the absence of PknD (Figure 5J). Thus, PknD promotes brain invasion by increasing attachment but not crossing, which it slightly deters. Furthermore, the PknD mutant, complement, and wild type crossed through ZO-1-ringed gaps of similar sizes (Figures 5K–5O). However, the dynamics of gap formation differed, with PknD mutant gaps remaining open much longer than wild type (Figure 5P, mean 45.8 min compared to 11.5 min), perhaps due to the lack of F-actin accumulation (compare Figures 2E and 5K). This suggests that F-actin accumulation is associated with gaps closing more quickly for the wild type and less quickly for the PknD mutant, allowing bacteria to cross more easily. Our findings are consistent with the appreciation that tight junction permeability depends on optimal interaction with F-actin, as both too weak and too strong associations diminish tight junction integrity.^50^ Thus, the F-actin rearrangements that aid attachment paradoxically counteract the tight junction reorganization required for crossing.

In sum, PknD promotes brain invasion by inducing F-actin rearrangements that help attach mycobacteria to endothelial cells. Importantly, our findings show that attachment and junctional remodeling are mediated by distinct mycobacterial functions.

### Cell surface determinants shared with nonpathogenic mycobacteria remodel junctions

Our findings suggested a model where remodeling is promoted by a surface-exposed or secreted factor from mycobacteria attached to the endothelium. To determine the importance of these factors, we used *M*. *marinum* killed by γ-irradiation, which are structurally intact but incapable of active protein secretion. Given that killed bacteria are rapidly destroyed by macrophages, we used macrophage-depleted larvae infected with similar numbers of live or γ-irradiated *M*. *marinum* and found equivalent brain infection (Figures S4A–S4C). We found that small clumps of γ-irradiated *M*. *marinum* attached to the microvasculature, crossed as efficiently as live microcolonies (Figure S4D), and were of a similar size (mean volumes, live 181 μm^3^ and γ-irradiated 151 μm^3^). Like live *M. marinum*, crossing clumps were larger than attached clumps (mean volumes, crossing 386 μm^3^ and attached 64 μm^3^), supporting the hypothesis that microcolony size increases crossing (Figure S4E). Similar to live bacteria, attachment was associated with F-actin recruitment (Figures S4F and S4G) followed by crossing through ZO-1-ringed gaps (mean diameter 4.8 μm) (Figures S4H–S4K). Thus, mycobacterial surface determinants, rather than active protein secretion, cause tight junction remodeling.

While both pathogenic and environmental mycobacterial species share conserved, complex cell walls, pathogenic species have some specific surface-associated determinants. Phthiocerol dimycoceroserate (PDIM) was an attractive candidate as it perturbs epithelial cell membranes.^51,52^ However, when we tested *Mycobacterium smegmatis*, the prototypical nonpathogenic, environmental mycobacterium that lacks PDIM,^37^ we found that it behaved identically to *M*. *marinum,* with equivalent brain bacterial burden and similar numbers of microcolonies attaching to and crossing the BBB (Figures S4L–S4O). The attachment was associated with F-actin rearrangements (Figures S4P and S4Q), and crossing invariably occurred through gaps surrounded by ZO-1 (Figures S4R and S4S). These findings led to the conclusion that junctional remodeling is induced by one or more surface factors shared between pathogenic (*M*. *tuberculosis* and *M*. *marinum*) and saprophytic (*M*. *smegmatis*) mycobacteria.

### Cyclopropanated TDM promotes invasion by increasing attachment and remodeling junctions

**Figure 6.**
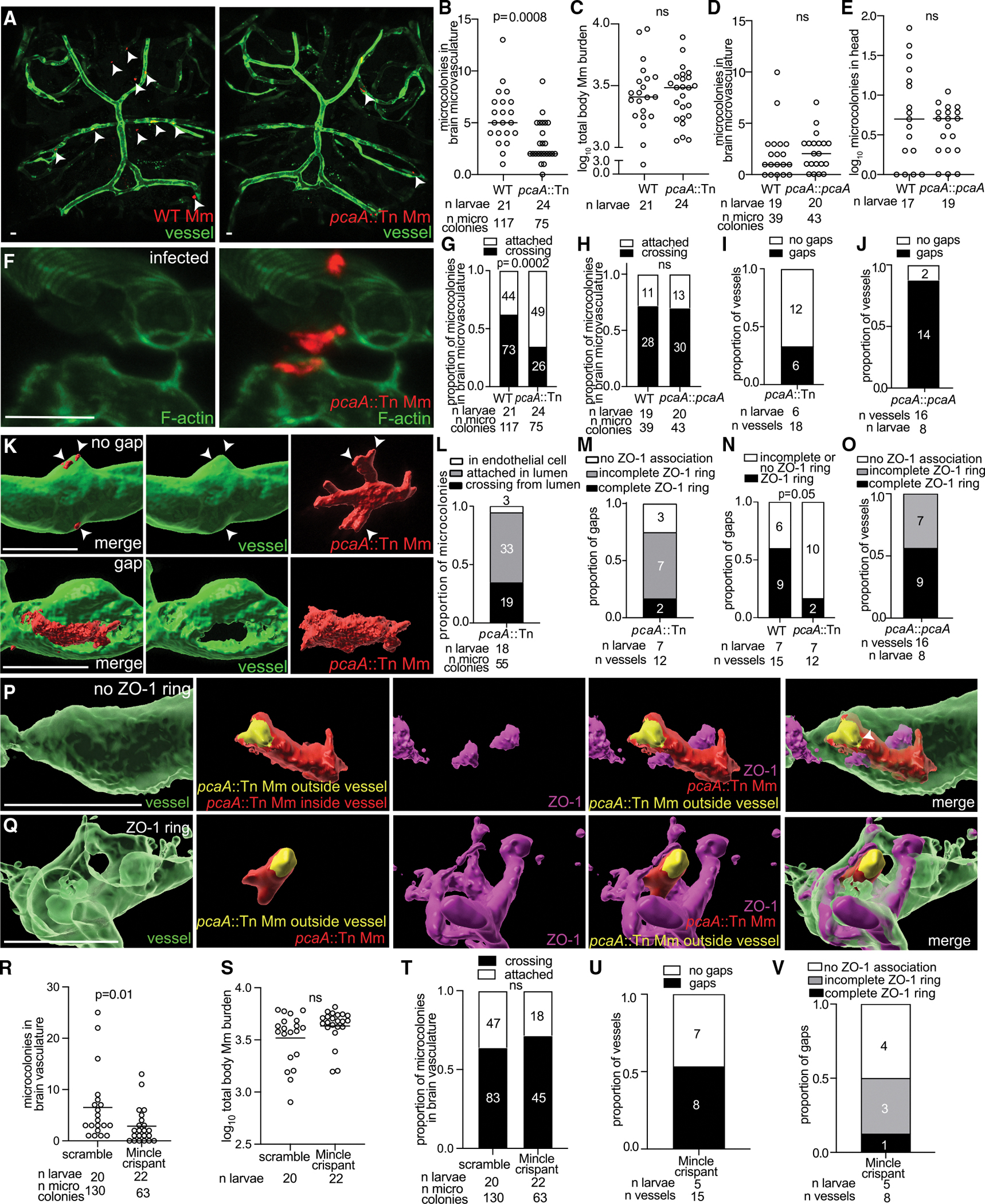
TDM recognition induces junctional remodeling (A) Representative confocal images of green-fluorescent brain vasculature in 2 dpi larvae infected with ~1000 CFU wild-type (WT) *M. marinum* (Mm) (left), or ~1,500 CFU *pcaA*::Tn Mm (right). Scale bars, 10 μm. (B) Total WT and *pcaA*::Tn Mm microcolonies in the brain vasculature (attached and crossing microcolonies). Horizontal bars, means; Student’s *t* test. Representative of 2 independent experiments. (C) WT or *pcaA*::Tn Mm burden per larva at 2 dpi quantified by fluorescent pixel counts (FPC) from the experiment in (A). Horizontal bars, means; ns, not significant; Student’s *t* test. Representative of 2 independent experiments. (D) Total WT and *pcaA*::Tn::*pcaA* Mm microcolonies in the brain vasculature (attached and crossing microcolonies). Horizontal bars, means; Student’s *t* test. (E) WT or *pcaA*::Tn::*pcaA* Mm burden per larva at 2 dpi quantified by FPC. Horizontal bars, means. (F) Representative confocal images of red-fluorescent *pcaA*::Tn Mm-infected vessels from *flk:GAL4;UAS:LifeAct-GFP* larvae at 2 dpi. Scale bars, 10 μm. (G) Proportion of attached or crossing WT and *pcaA*::Tn Mm microcolonies (excluding microcolonies in the brain) in brain blood vessels from larvae in (A). Fisher’s exact test. Representative of 2 independent experiments. (H) Proportion of attached or crossing WT and *pcaA*::Tn::*pcaA* Mm microcolonies (excluding microcolonies in the brain) in blood vessels. Fisher’s exact test; ns, not significant. (I) Proportion of vessels with or without gaps in *pcaA*::Tn Mm infected vessels. Representative of 2 independent experiments. (J) Proportion of vessels associated with gaps in *pcaA*::Tn:*pcaA* Mm infected vessels. (K) 3D rendered, representative confocal image of 3 dpi larvae with green-fluorescent blood vessels, infected with red-fluorescent *pcaA*::Tn Mm. Top, *pcaA*::Tn Mm microcolony crossing blood vessel without apparent gap (arrowheads). Bottom, *pcaA*::Tn Mm microcolony crossing through a gap. Scale bars, 10 μm. (L) Proportion of vessels containing *pcaA*::Tn Mm that are attached, crossing, or inside of an endothelial cell. (M) Proportion of gaps associated with a complete, incomplete, or no ZO-1 ring for *pcaA*::Tn Mm microcolonies that are crossing blood vessels. (N) Proportion of WT or *pcaA*::Tn Mm associated gaps associated with a complete or incomplete/no ZO-1 ring. WT data from Figure 4C; Fisher’s exact test. (O) Proportion of gaps associated with a complete, incomplete, or no ZO-1 ring for *pcaA*::Tn::*pcaA* Mm microcolonies that are crossing blood vessels. (P and Q) 3D rendered, representative confocal images from a 3 dpi larva with green-fluorescent vessels infected with red-fluorescent *pcaA*::Tn Mm, fixed and stained with anti-ZO-1 antibody (pseudo-colored magenta). *pcaA*::Tn Mm microcolony without associated gap or ZO-1 ring (P). *pcaA*::Tn Mm microcolony with associated gap, partially ringed by ZO-1 (Q). Yellow indicates parts of the microcolony that have exited the vasculature and entered the brain. Scale bars, 10 μm. (R) Total Mm microcolonies in the brain vasculature (attached and crossing microcolonies) in WT (scramble) and Mincle crispant larvae. Horizontal bars, means; Student’s *t* test. Representative of 2 independent experiments. (S) WT or *pcaA*::Tn Mm burden per larva at 2 dpi quantified by FPC from experiment in (R). Horizontal bars, means; ns, not significant; Student’s *t* test. Representative of 2 independent experiments. (T) Proportion of attached or crossing Mm microcolonies (excluding microcolonies in the brain) in blood vessels from WT or Mincle crispant larvae. Fisher’s exact test; ns, not significant. Representative of 2 independent experiments. (U) Proportion of vessels associated with blood vessel gaps in Mm-infected vessels from Mincle crispant larvae. Representative of 2 independent experiments. (V) Proportion of gaps associated with a complete, incomplete, or no ZO-1 ring for microcolonies that are crossing blood vessels in Mincle crispant larvae.

Another strong candidate was TDM, an abundant outer cell wall glycolipid that is present in *M*. *tuberculosis*, *M*. *marinum*, and *M*. *smegmatis*.^53–55^ Cyclopropanated TDM is important for the characteristic cording morphology of mycobacteria, which has been recently linked to the ability of *M*. *tuberculosis* to penetrate between alveolar epithelial cells *in vitro*.^53,56^ Independent of cording, cyclopropanated TDM engages several eukaryotic signaling pathways,^56–59^ such as VEGF-mediated angiogenesis in zebrafish.^54^ We tested an *M*. *marinum* transposon mutant in PcaA, the cyclopropane synthase that *cis*-cyclopropanates TDM at the proximal position.^53,54^ Despite equivalent overall bacterial burden to wild type, the PcaA mutant had reduced brain dissemination (Figures 6A–6C), which was reversed by complementation (Figures 6D and 6E). Similar to the PknD mutant, attachment by the PcaA mutant was not associated with F-actin rearrangements, indicating an attachment defect (Figure 6F). However, in contrast to the PknD mutant, the PcaA mutant exhibited a crossing defect over and above its attachment defect. Fewer microcolonies crossed the BBB (Figure 6G), which was reversed by complementation (Figure 6H). Thus, while PknD promotes attachment, PcaA promotes both attachment and crossing. Strikingly, only 33% of PcaA mutant microcolonies crossed through obvious gaps, compared to nearly all in wild type and the PknD mutant (Figures 6I and 6J). While most PcaA mutant microcolonies protruded into the brain without an obvious gap (Figure 6K, compare top and bottom panels), they were still likely crossing between junctions rather than by transcytosis, as the bacteria emerging from the vessel had no endothelial membrane (green fluorescence) surrounding them (Figure 6K, arrowheads). Furthermore, as with wild-type bacteria, only a minority (5%) of PcaA mutant microcolonies were found inside endothelial cells, none of which were crossing (Figure 6L). Finally, PcaA mutants crossing through gaps often had absent or incomplete ZO-1 rings, which were complete in only 17% of instances, compared to 60% for wild type or complement (Figures 6M–6Q).

PcaA mutant microcolonies did not have the corded growth phenotype seen in wild-type bacteria (Figures S5A–S5C). To clarify if the attachment and crossing defects of the PcaA mutant were due to TDM or its defect in cording, we tested brain invasion by Erp mutant *M*. *marinum,* which has functional TDM but deficient cording.^60^ The Erp mutant attached to the brain microvasculature and accumulated F-actin as frequently as wild type in equivalently infected larvae (Figures S5D–S5F). The majority of these microcolonies crossed from the vessel lumen through gaps without infecting endothelial cells (Figures S5G–S5I). Like wild type, the majority of gaps were associated with a ZO-1 ring (Figures S5J and S5K). These findings suggest that cording does not contribute to attachment, F-actin rearrangements, or remodeling of ZO-1-ringed gaps.

In sum, cyclopropanated TDM is necessary for brain microvascular endothelial F-actin rearrangements that increase mycobacterial attachment as well as promote tight junction remodeling manifested by openings in the endothelium surrounded by ZO-1. In its absence, individual members of attached microcolonies can still cross by squeezing between the junctions. However, the overall reduction in brain invasion in the PcaA mutant suggests that this process is not as efficient as when the junctions can be remodeled to create gaps.

TDM is a potent immunostimulatory glycolipid that is recognized by the C-type lectin, Mincle, expressed on the surface of myeloid cells.^61^ Mice deficient for Mincle have significantly diminished macrophage activation and fail to form TDM-induced lung granulomas.^57^ Mincle is also expressed on human and mouse brain endothelial cells.^62,63^ Therefore, we surmised that TDM recognition by Mincle expressed by endothelial cells could be involved in mycobacterial brain invasion. To test this, we generated G0 Mincle crispants. The intravenous injection of *M*. *marinum* produced an infection phenotype similar to PcaA mutant infection in wild-type larvae, even with comparable overall bacterial burdens. Specifically, we observed reduced dissemination to the brain (Figures 6R and 6S), impaired gap-associated crossing of the brain microvasculature (compare Figures 3C; 6T, 6U, and S6A), and absent or incomplete ZO-1 rings surrounding the gaps that formed (Figures 6V, S6B, and S6C). Thus, TDM recognition by Mincle interactions facilitates gap formation and crossing of the brain microvasculature.

### *M. tuberculosis* invades the brain via attachment and junctional remodeling, with conserved roles for PknD and PcaA

**Figure 7.**
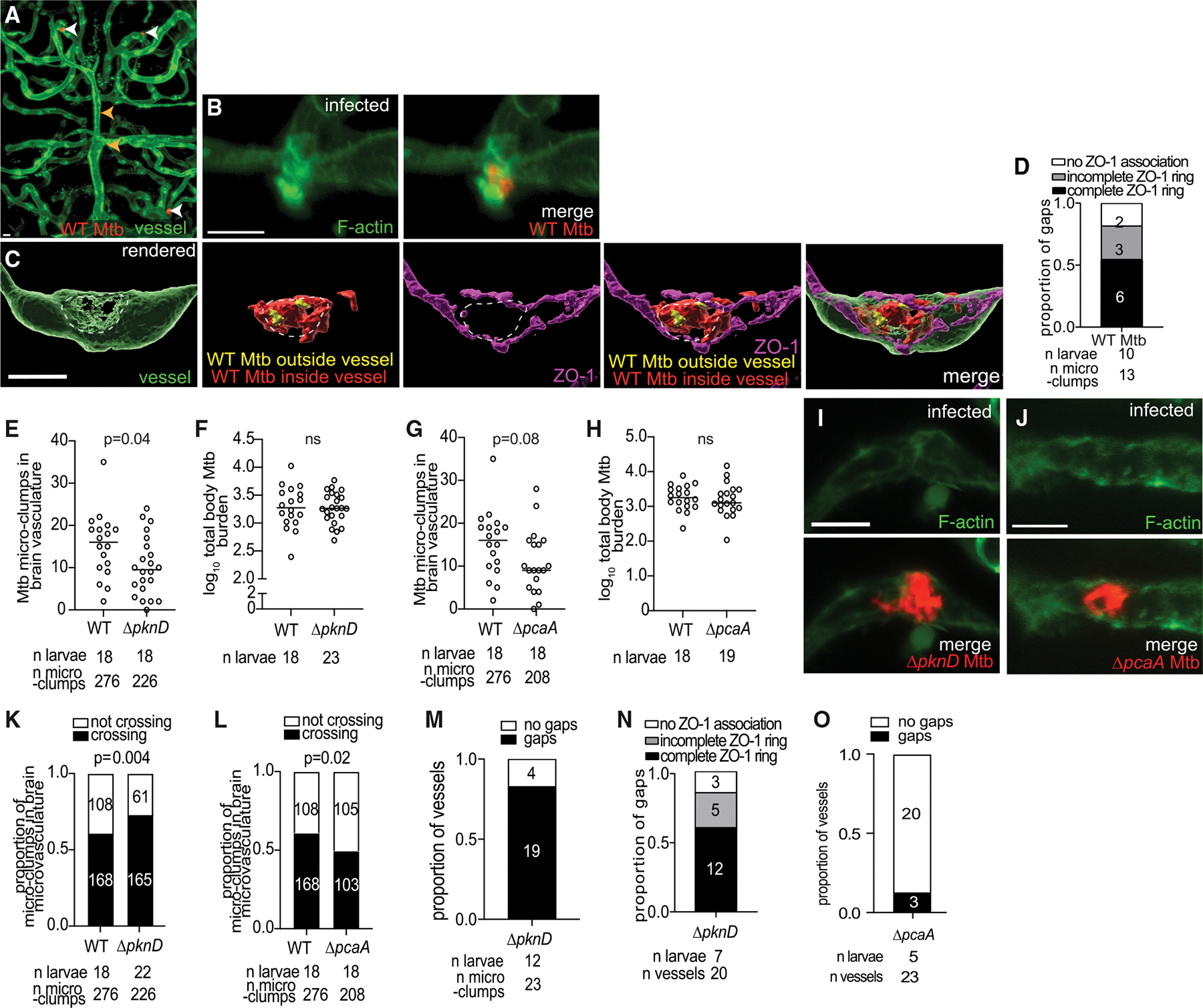
*M. tuberculosis* crosses the blood-brain barrier through ZO-1 rings (A) Representative confocal image of the green-fluorescent brain vasculature in a 3 dpi larvas infected with ~100 CFU red-fluorescent mc^2^6206 *M. tuberculosis* (Mtb). White arrowheads, Mtb in blood vessels; orange arrowheads, Mtb in brain parenchyma. Scale bar, 10 μm. (B) Representative confocal image of infected vessels from a *flk:GAL4;UAS:LifeAct-GFP* larva infected at 3 dpi with ~300 CFU red-fluorescent Mtb, showing green-fluorescent F-actin accumulation around the micro-clump. Scale bar, 10 μm. (C) 3D rendered, representative confocal image of a 3 dpi larva with green-fluorescent vessels infected with ~300 CFU red-fluorescent Mtb, fixed, and stained with anti-ZO-1 antibody (pseudo-colored magenta). Dashed circle, gap ringed by ZO-1 under Mtb micro-clump. Yellow indicates parts of the micro-clump that have exited the vasculature and entered the brain. Scale bar, 10 μm. (D) Proportion of gaps associated with a complete, incomplete, or no ZO-1 ring for Mtb micro-clumps that are crossing blood vessels. (E) Total WT and Δ*pknD* Mtb micro-clumps in the brain vasculature (attached and crossing micro-clumps). Horizontal bars, means; Student’s *t* test. (F) WT or Δ*pknD* Mtb burden per larva at 3 dpi quantified by fluorescent pixel counts (FPC) infected with ~300 CFU. Horizontal bars, means; Student’s *t* test; ns, not significant. (G) Total WT and Δ*pcaA* Mtb micro-clumps in the brain vasculature (attached and crossing). Horizontal bars, means; Student’s *t* test. (H) WT or Δ*pcaA* Mtb burden per larva at 3 dpi quantified by FPC infected with ~300 CFU. Horizontal bars, means; Student’s *t* test; ns, not significant. (I) Representative confocal image of a red-fluorescent Δ*pknD* Mtb-infected vessel from *flk:GAL4;UAS:LifeAct-GFP* larvae at 4 dpi. Scale bar, 10 μm. (J) Representative confocal image of a red-fluorescent Δ*pcaA* Mtb-infected vessel from *flk:GAL4;UAS:LifeAct-GFP* larvae at 4 dpi. Scale bar, 10 μm. (K and L) Proportion of attached or crossing WT and Δ*pknD* Mtb (K) or WT and Δ*pcaA* Mtb (L) micro-clumps (excluding micro-clumps in the brain) in brain blood vessels. Fischer’s exact test. (M) Proportion of infected vessels associated with gaps in Δ*pknD* Mtb-infected larvae. (N) Proportion of gaps associated with a complete, incomplete, or no ZO-1 ring for Δ*pcaA* Mtb clumps that are crossing blood vessels. (O) Proportion of infected vessels associated with gaps in Δ*pcaA* Mtb-infected larvae.

To see if *M. tuberculosis* also invades the brain paracellularly, we used a fluorescently labeled double leucine and pantothenic acid auxotrophic strain of *M. tuberculosis*, mc^2^6206, which can be safely handled in our biosafety level 2 microscopy suite.^64^ Injection of *M. tuberculosis* mc^2^6206 intravenously into macrophage-depleted larvae showed that it appeared in the brain microvasculature within days (Figure 7A). Similar to *M. marinum*, attachment of pre-existing clumps was associated with F-actin recruitment (Figure 7B). Neither internalization nor transcytosis was observed for 23 attached clumps, of which 12 were in the process of crossing. Instead, as with *M. marinum*, crossing occurred through ZO-1-ringed gaps of a similar size (mean diameter 6.6 μm) and morphology to those with *M. marinum* (Figures 7C and 7D).

Next, to verify if *M. tuberculosis* uses PknD and PcaA for attachment and crossing, we tested the corresponding *M. tuberculosis* mutants. Like their *M. marinum* counterparts, the *M. tuberculosis* PknD and PcaA mutants both had reduced attachment to the brain microvasculature and failed to mediate F-actin rearrangements (Figures 7E–7J). Consistent with *M. marinum*, the *M. tuberculosis* PknD mutant only had an attachment defect, whereas the PcaA mutant had both an attachment and crossing defect. Among attached clumps, the *M. tuberculosis* PknD mutant crossed slightly better, similar to *M. marinum*; in contrast, the attached PcaA clumps crossed less frequently (Figures 7K and 7L). Consistent with these phenotypes, the PknD mutant crossed through ZO-1-ringed gaps (Figures 7M and 7N), while few PcaA mutant crossings were associated with gaps (2/23) (Figure 7O). Thus, *M. tuberculosis* invades the brain using the same paracellular mechanism identified for *M. marinum.*

### *M. smegmatis* PcaA is required for junctional remodeling

The finding that a cell surface lipid shared between *M. marinum* and *M. smegmatis* had brought us to the discovery that cyclopropanated TDM disrupts tight junctions to enable mycobacterial brain invasion. This suggests that *M. smegmatis* also uses PcaA to cyclopropanate TDM and mediate junctional reorganization. The *M. smegmatis* PcaA homolog (MSMEG_1351) has been shown to *cis*-cyclopropanate α-mycolic acids and restore both α-mycolic acid *cis*-cyclopropanation and cording in an *M. bovis* BCG PcaA mutant.^55^ To test this, we created an *M. smegmatis* PcaA mutant. *M. smegmatis* does not exhibit as strong cording as *M. marinum* and *M. tuberculosis*; the *M. smegmatis* PcaA mutant nevertheless showed the expected reduction in cording (Figure S5C). Like the *M. marinum* and *M. tuberculosis* PcaA mutant strains, the *M. smegmatis* PcaA mutant had reduced numbers in the brain microvasculature, decreased invasion, and attached colonies without F-actin rearrangements (Figures S7A–S7D). Crossing was reduced and was not associated with tight junction openings (Figures S7E–S7F). Rather, the bacteria often crossed through imperceptible gaps in the tight junctions that did not have ZO-1 rings (Figures S7G–S7I). Thus, PcaA can mediate brain microvasculature traversal in both pathogens and saprophytes.

## DISCUSSION

The use of time-lapse microscopy in the transparent zebrafish larva has provided sequential, granular details of the very first and most elusive step of TB meningitis, how mycobacteria invade the brain. Our work brings into question the long-standing dogma of the “Trojan horse” model that macrophages carry mycobacteria into the brain vasculature and into the brain. Instead, free mycobacteria enter the brain microvasculature where they attach to endothelial membranes, by inducing endothelial cell F-actin rearrangements and then invade by inducing dynamic junctional reorganization that results in transient gaps through which the bacteria enter the brain. The two mycobacterial mutants—PknD and PcaA—enable separation of the distinct roles of the F-actin rearrangements and junctional reorganization in the invasion process. TDM is required for both attachment and crossing through its receptor Mincle, which is associated with F-actin rearrangements and tight junction reorganization. In contrast, PknD is required only for F-actin rearrangements and attachment, but is dispensable for the reorganization of tight junctions. Those PknD mutant microcolonies that do manage to attach in the absence of F-actin rearrangements can remodel junctions and cross through the ensuing gaps. Thus, mycobacteria mediate tight junction reorganization independently of actin cytoskeleton rearrangements, whereas these are linked under homeostatic conditions.^65^ Indeed, our findings with the PknD mutant show that F-actin cytoskeletal rearrangements can impede crossing.

Although we saw a dramatic rearrangement of ZO-1, we cannot exclude that TDM’s direct interactions are with other or additional proteins, either from among the tight junction complex that in turn causes ZO-1 reorganization or from other linked junctional complexes, such as the adherens junctions.^65^ Another unresolved issue is how cyclopropanated TDM reorganizes junctions. One possibility is that it mediates the cording morphology that causes physical disruption of junctions, as has been recently proposed.^56^ A second is that it acts as a signaling molecule on one or more tight junction proteins, which would be consistent with its role in signaling in a variety of eukaryotic processes.^56–59^ Cording and signaling could work in concert, with the corded morphology apposing bacterial microcolonies and the endothelium, to optimize signaling. Our findings that γ-irradiated *M. marinum*, Δ*erp M. marinum*, and *M. smegmatis* have less corded morphology than wild-type *M. marinum* and *M. tuberculosis*, which cross through ZO-1-ringed membrane disruptions, suggest that cyclopropanated TDM-mediated signaling contributes to tight junction reorganization, over and above contributing to cording.

In previous work using HBMECs to study the role of PknD, the authors, upon observing PknD-mediated actin polymerization, attachment, and internalization, reasonably surmised that PknD promotes bacterial transcytosis.^27^ Subsequently published findings explain the discrepancy with our findings—transcytosis is greatly downregulated *in vivo* through interactions of the BBB endothelium with pericytes in the brain, including in the zebrafish larvae.^8,29,66,67^ PknD has been proposed to bind endothelial cell laminin α2 through its sensor domain,^27^ but it can also phosphorylate proteins involved in cell wall transport through its kinase domain.^68^ The association of laminin α2 with the basolateral rather than luminal surface of blood vessels rules out that it uses this interaction for tight binding. Rather, PknD likely modifies the mycobacterial cell surface to promote attachment. That TDM, another cell surface modifier, is also necessary for F-actin rearrangements and tight attachment supports this model.

Our finding that mycobacteria cross in vivo predominantly by paracellular transit is striking, as among other meningeal pathogens, only *N. meningitidis* has a predominantly paracellular mechanism.^1,9^ Similar to our observations for mycobacteria, *N. meningitidis* also adheres to the endothelium and forms microcolonies. However, the mode of tight junction disruption appears to be distinct for the two pathogens. The attached *N. meningitidis* microcolony recruits both cellular actin and multiple junctional proteins to it.^31,69^ This sequestration of junctional proteins away from the junctions makes the junctions leaky.^31,69,70^ Indeed, our finding that the PknD mutant remodels tight junctions without causing F-actin rearrangements demonstrates that mycobacteria have a distinct mechanism.

Another unique feature of mycobacteria’s paracellular transit is that it occurs through dynamic junctional remodeling, creating only transient gaps that seal quickly. This finding explains how TB meningitis is a two-step event with mycobacterial invasion into the brain first causing granuloma formation and meningitis occurring only months later, if and when these granulomas erode into the meninges. The host can be asymptomatic in the intervening period. A mystery has been how mycobacteria could invade the brain in the first place without causing consequential BBB disruption that would be clinically apparent. Our finding that the disruptions are transient, allowing mycobacteria to invade while leaving the BBB intact, provides the answer. Furthermore, we find that the invading mycobacteria initiate Rich foci by attracting and infecting microglia. This again addresses the two-step model, as to how tuberculous granulomas might form in the brain in the first place, without causing too much inflammation. It uses the already available macrophages in the brain.

Like many important mycobacterial virulence factors, cyclopropanated TDM is present in nonpathogenic mycobacteria, where it likely provides environmental protection by forming the bacteria into multicellular communities.^55^ *In vivo* work has linked mycobacterial cording to increased mycobacterial growth by inhibiting re-phagocytosis into macrophages, which can be growth restricting.^71^ It is striking that *M. tuberculosis* invades the brain using this virulence determinant shared with nonpathogens. From an evolutionary standpoint, this is not surprising since meningitis caused by any bacterium is an accidental dead end, providing no benefit to the bacterium in terms of transmission and thereby evolutionary survival. This is also the case for *M. tuberculosis*, where only the pulmonary form is transmissible. Thus, the bacterial factors that cause meningitis in all cases have evolved for other purposes; in the case of meningitis caused by commensal pathogens, these have been described as colonization factors that run amok.^37^ Our work extends this paradigm to the obligate pathogen *M. tuberculosis* to show that determinants that clearly evolved for environmental survival are responsible for the deadliest form of disease caused by humanity’s greatest killer.^20^

### Limitations of the study

Our study demonstrates the mechanism by which mycobacteria breach the BBB in zebrafish larvae. While it has been demonstrated previously that *M. marinum* causes a TB-like infection in zebrafish, we recognize that mycobacterial pathogenesis in a mammalian system may present differently. However, there are several factors that support the translatability of zebrafish infection models to human or mammalian disease: (1) ~70% of human genes have known zebrafish orthologs; (2) the innate immune responses are similar between humans and zebrafish; (3) granulomas in zebrafish resemble those found in human TB; (4) *lta4h* is a susceptibility locus for *M. marinum* disease in zebrafish, as it is for TB in humans, and (5) both PknD and PcaA are important for mycobacterial virulence in mammals, and PknD for brain infection. Nevertheless, future study is required to confirm if mycobacteria use the Mincle-TDM pathway to disrupt endothelial junctions to cross into the brain in mammals.

In addition to dissecting the mechanism of *M. marinum* brain invasion in zebrafish, we confirmed our findings with *M. tuberculosis*, the causative agent of tuberculous meningitis in humans. For biosafety requirements, we utilized the double auxotrophic *M. tuberculosis* strain, mc^2^6206, instead of the more virulent parent strain, H37Rv. The mc^2^6206 and the H37Rv strains have been shown to behave similarly in several ways, displaying similar growth rates, both *in vitro* and within macrophages, and responding similarly to anti-TB agents. However, mc^2^6206 has also been shown to display an increased stress response and can induce higher cytokine and chemokine responses in immune cells compared to H37Rv. Therefore, how the H37Rv *M. tuberculosis* strain behaves in this system remains to be seen.

## RESOURCE AVAILABILITY

### Lead contact

Further information and requests for resources and reagents should be directed to and will be fulfilled by the lead contact, Cressida A. Madigan (cmadigan@ucsd.edu).

### Materials availability

Zebrafish lines and bacterial strains generated in this study are available from the lead contact.

### Data and code availability

- All data reported in this paper will be shared by the lead contact upon request.
- This paper does not report original code.
- Any additional information required to reanalyze the data reported in this paper is available from the lead contact upon request.

## STAR★METHODS

Detailed methods are provided in the online version of this paper and include the following:

### EXPERIMENTAL MODEL AND STUDY PARTICIPANT DETAILS

#### Zebrafish husbandry and infections

Zebrafish husbandry and experiments were conducted in compliance with guidelines from the U.S. National Institutes of Health and approved by the University of California San Diego Institutional Animal Care and Use Committee and the Institutional Biosafety Committee of the University of California San Diego. Wildtype AB strain zebrafish or transgenics in the AB background were used, including Tg(*kdrl:GFP*),^30^ Tg(*fliE:GAL4;UAS:dsRed*),^76^ Tg(*fliE:GFP*),^76^ Tg(*flk:GFP*),^77^ Tg(*flk:GAL4;UAS:Lifeact-GFP*),^74^ Tg(*mpeg1:dsRed*),^35^ Tg(*flt1:tomato*),^75^ Tg(*flk:alpha-catenin-GFP*),^78^ and Tg(*flk:moesin-GFP*).^78^ Larvae were anesthetized with 2.8% Syncaine (Syndel #886-86-2) prior to imaging or infection. Larvae of indeterminate sex were infected by injection of 10 nL into the caudal vein at 3 days post fertilization (dpf) using a capillary needle containing bacteria diluted in PBS + 2% phenol red (Sigma #P3532), as previously described.^72^ Titered, single-cell suspensions were prepared for all *M. marinum* strains prior to infection by passing cell pellets from mid-log phase cultures (OD_600_ 0.5 ± 0.1) repeatedly through a syringe to remove clumps, as described.^72^ After caudal vein injections were done, the same needle was used to inject onto 7H10 (Sigma-Aldrich #M199) agar plates containing 50 μg/mL hygromycin B (Thermofisher #10687010) or 50 μg/mL kanamycin (TCI #K0047) in triplicate to determine colony forming units (CFUs) of the inoculum. When two different bacterial strains were compared for bacterial burden directly, several groups of larvae (*n*=20 or more) were infected with different inocula of each strain. On the day of the comparison, equivalently infected groups of larvae were determined by FPC, as described,^72^ to assure the comparison was not biased by in vivo growth differences between the two strains. ~100–500 CFUs of wildtype *M. mainum* were administered to the larvae for experiments unless otherwise specified. After infection, larvae were housed at 28.5°C, in fish water containing ddH_2_O, 14.61 g/L sodium chloride (JT Baker #3628-F7), 0.63 g/L potassium chloride (Sigma-Aldrich #P3911), 1.83 g/L calcium chloride (G-Biosciences #RC-030), 1.99 g/L magnesium sulfate heptahydrate (MP Biomedicals #194833), methylene blue chloride (Millipore Sigma #284), and 0.003% 1-phenyl-2-thiourea (PTU, Sigma-Aldrich #189235) to prevent melanocyte development. To generate G0 Mincle crispants, guide RNAs were prepared by combining equimolar concentrations of Alt-R CRISPR-Cas9 tracrRNA (IDT #1072532) with *mincle* crRNA (IDT; sequences listed in the key resources table below) or Alt-R Negative Control crRNA (IDT #1072545) in nuclease-free Duplex Buffer (IDT #11-01-03-01) at 95°C for 5 min. Alt-R S.p. Cas9 Nuclease V3 (IDT #1081058) was diluted to a working concentration of 0.5 μg/μL in Cas9 dilution buffer (20 mM HEPES; 150 mM KCI, pH 7.5) and then combined with the prepared gRNAs at equimolar concentrations and heated at 37°C for 10 min to create the ribonucleoprotein (RNP) complexes. Larvae of indeterminate sex were injected with either 5 nL of the *mincle* or negative control RNP complexes at the single-cell stage. After imaging, larvae were individually sacrificed to collect genomic DNA and the *mincle* gene was PCR-amplified and Sanger-sequenced to verify gene disruption.

### METHOD DETAILS

#### Bacterial strains

*M. marinum* M strain (ATCC #BAA-535), mutants strains (*pknD*::Tn6042, Δ*esx1, pcaA*::Tn20324, Δ*erp*), and complemented strains (*pknD*::Tn6042:*pknD* and *pcaA*::Tn20324:*pcaA*) expressing tdTomato, wasabi, cerulean, or eBFP2 under control of the msp12 promoter,^72,81^ were grown in 50 μg/mL hygromycin B (ThermoFisher, #10687010) or 50 μg/mL kanamycin (TCI, #K0047) in liquid culture, consisting of 7H9 Middlebrook medium (Sigma-Aldrich, #M0178) supplemented with 2.5% oleic acid (Sigma, #O1008), 50% glucose, and 20% Tween-80 (Sigma, #P1754).^72^ Agar plates contained 7H10 Middlebrook agar (HiMedia, #M199), supplemented with oleic acid, albumin (Sigma, #A9647), dextrose, and Tween-80.^72^ To transform *M. marinum pknD*::Tn6042 with pmsp12:eBFP2, the bacterial pellet from a 10 mL liquid culture of *M. marinum* (OD_600_ 0.8) was collected by centrifugation at 4000 × g for 10 min at 4°C. After washing in 10 mL ice-cold 10% glycerol, the bacterial pellet was resuspended in 1 mL ice-cold 10% glycerol, and centrifuged at 7300 × g, for 2 min, at 4°C. The bacterial pellet was resuspended in 100 μL of ice-cold 10% glycerol. 1 μL containing 100 ng of pmsp12:eBFP2 was added to 30 μL of the bacterial resuspension, which was electroporated (800 Ω, 25 μF, 2.5kV) in a 2 mm sterile cuvette. Cells were recovered in 900 μL 7H9+OADS+Tween-80 for 24 h at 33°C. Cells were plated on 7H10 Middlebrook agar plates containing hygromycin B. Colonies were isolated by verifying for the presence of hygromycin B resistance, blue fluorescence, and the presence of the transposon at *pknD* (via Sanger sequencing; *pknD* F primer sequence: TAGCGTGAATATGTAGGGTC; *pknD* R primer sequence: ATCTACACCGAGCTCACCAA). The unlabelled *pknD*::Tn6042 was complemented by transforming with pJKS226 and isolated by verifying the presence of hygromycin B resistance, presence of the *pknD* gene and red fluorescence. For zebrafish larvae infection, ~1000 CFUs of Δ*esx*1 *M. marinum*, ~3000 CFUs of *pknD*::Tn *M. marinum*, or ~1500 CFUs of *pcaA*::Tn M*. marinum* were injected. Higher CFUs of the *pknd*::Tn and *pcaA*::Tn mutant were administered to the larvae compared to wildtype to ensure infection matching in vivo.

*M. tuberculosis* Δ*leuD*Δ*panCD* mc^2^ 6206 expressing tdTomato was grown at 37°C under hygromycin B selection in Middlebrook 7H9 medium (Sigma-Aldrich, #M0178) supplemented with oleic acid (Sigma, #O1008), albumin (Sigma, #A9647), dextrose (Sigma-Aldrich, #D9434-500g), Tween-80 (Sigma, #P1754), catalase (Sigma-Aldrich, #C1345-1G), 0.05 mg/mL L-leucine (Milipore, #4330-100GM), and 0.024 mg/mL calcium pantothenate (Sigma-Aldrich, # PHR1232). Δ*pcaA* and Δ*pknD* strains were constructed by recombineering as previously described.^79^ Briefly, pNit-SacB-Kan transformed mc^2^ 6206 was induced with 1 μM isovaleronitrile at OD_600_ = 0.8 for 8 h followed by addition of 0.2 M glycine and incubation at 37°C. Electrocompetent cells were prepared (at room temperature) and transformed with ~1 μg PCR-purified DNA fragments from an EcoRV digest of pJKS181 (PknD) and pJKS217 (PcaA) at 37°C for 16 h. Transformants were electroporated (1000 Ω, 25 μF, 2.5kV) in a 2 mm sterile cuvette and cells recovered in 2 mL 7H9 + OADC + Tween-80 + 50 μg/mL L-leucine + 24 μg/mL D-pantothenic acid for 24 h at 37°C, then plated on supplemented 7H10 Middlebrook agar plates containing 50 μg/mL hygromycin B. After 4–6 weeks hygromycin resistant isolates were screened for correct insertion of the deletion cassette by sequencing PCR products spanning the junction between the integrated knockout construct and flanking genome regions. For zebrafish larvae infection, ~300 CFUs of *M. tuberculosis* were injected.

γ-irradiated *M. marinum*-td:Tomato was made by irradiating 5 uL single-cell aliquots with 2000 Gy γ-irradiation using a JL Shepherd MK I Cesium-137 irradiator. γ-irradiated bacterial cells were confirmed non-viable by plating on 7H10 agar plates. For this reason, experiments involving γ-irradiated *M. marinum* do not have CFU counts listed.

*M. smegmatis* strain mc^2^ 155 was grown in liquid medium containing 50 μg/ml hygromycin B (ThermoFisher, #10687010) in 7H9 Middlebrook medium (Sigma-Aldrich, t# M0178) supplemented with 2.5% oleic acid (Sigma, #O1008), 50% glucose, and 20% Tween-80 (Sigma, #P1754).^72^ Agar plates were 7H10 Middlebrook agar (HiMedia, #M199) supplemented with 2.5% oleic acid (Sigma, #O1008), 50% glucose, and 20% Tween-80 (Sigma, #P1754).^72^ The Δ*pcaA* (*MSMEG_1351*) strain was constructed by ORBIT as previously described.^80^ Briefly, pKM461 transformed mc^2^ 155 was induced with 500 ng/mL ATc at OD_600_ = 0.6 for 4 h and incubated at 37°C. Electrocompetent cells were prepared and transformed with 1 μg MSMEG_1351del ultramer and ~300 ng pJKS146. Transformants were electroporated (1000 Ω, 25 μF, 2.5kV) in a 2 mm sterile cuvette and cells recovered in 2 mL 7H9 + OADS + Tween-80 overnight then plated on 7H10 Middlebrook agar plates containing 50 μg/mL hygromycin B. After 7 days hygromycin resistant isolates were screened for insertion of the deletion cassette by PCR. The deletion cassette was excised by transforming deletion mutants with pCre-SacB-Zeo, isolating zeocin resistant colonies and screening for excision of the deletion cassette by PCR of the region followed by sequencing. *M. smegmatis* strains were grown in liquid culture (OD_600_ = 0.8) and transformed as described above with pTEC27.^72,81^ Transformants were isolated by verifying the presence of hygromycin B resistance and red fluorescence. For zebrafish larvae infection, ~500 CFUs of *M. smegmatis* were injected.

#### Plasmid construction

For ORBIT, pJKS146 was constructed by Q5 mutagenesis of pKM464 with primers JS285 and JS286. For recombineering plasmids, pKM342 (Addgene #71486) was domesticated by Q5 mutagenesis to remove the BsaI sites. The hygromycin cassette was amplified with JS362 and JS363 and the vector backbone was amplified with JS358 and JS359. The upstream flanking region of *pknD* was amplified with JS364 and JS365. The downstream flanking region of *pknD* was amplified with JS360 and JS361. The vector, upstream, downstream and hyg fragments were assembled by Golden Gate assembly to generate pJKS181. The upstream flanking region of *pcaA* was amplified with JS446 and JS447. The downstream flanking region of *pcaA* was amplified with JS444 and JS445. The vector, upstream, downstream and hyg fragments were assembled by Golden Gate assembly to generate pJKS217. For complementation, *M. marinum pknD* was amplified with JS487 and JS488 and cloned by IVA^82^ into pMV261 (Novopro V012795) linearised with JS485 and JS486. *hsp60*:*pknD* was amplified from the resulting vector with JS495 and JS496 and cloned by IVA into pTEC27, linearised by JS493 and JS494, to generate pJKS226.

#### Monocyte depletion, fluorospheres, and stains

Macrophage depletion was accomplished by morpholinos or clodronate-loaded liposomes (Liposoma #C-005). *pu.1* morpholinos were designed to the transcription initiation site (CCTCCATTCTGTACGGATGCAGCAT) and the exon 4–5 boundary (GGTCTTTCTCC TTACCATGCTCTCC) and combined to final concentrations of 0.375 mM and 0.025 mM, respectively.^34^ Morpholinos were diluted in tango buffer (Thermo Scientific #BY5) containing 2% phenol red (Sigma-Aldrich #P3532) and injected into the yolk of 1–2 cell-stage embryos in 1 nL.^83^ Chlodronate liposomes (LC) or PBS^84^ were diluted 1:5 in PBS + 2% phenol red and injected in 10 nL into 2 dpf larvae via the caudal vein. LC depletion was done for all experiments involving *M. tuberculosis, Δesx-1 M. marinum*, *pknD*::Tn *M. marinum*, γ-irradiated *M. marinum*, *M. smegmatis*, and *pcaA*::Tn *M. marinum* and Mincle crispants. For experiments involving 0.02 μm fluorospheres (Invitrogen #F8782), the reagent was diluted 1:10 in PBS and injected into the caudal vein on the day of imaging. To visualize vessels in larvae without transgenic fluorescent vessels, Alexa 647 Dextran (ThermoFisher, #D22914) or FITC-Dextran (Invitrogen, #D1820) was diluted 1:10 in PBS and injected into the caudal vein at the time of imaging. To visualize cell lysis, propidium iodide (Invitrogen #P3566), was diluted to 100 μg/mL in PBS and injected in 10 nL into the caudal vein on the day of imaging. To label monocytes that were in circulation before entering the brain, Hoechst (Invitrogen #H21486) was diluted to 100 μg/mL in PBS and injected in 10 nL into the caudal vein every day prior to imaging.

#### *α*-ZO-1 whole mount immunofluorescence

For ZO-1 immunohistochemistry, larvae were fixed in 1 mL 4% paraformaldehyde solution (PFA) (Fischer scientific #AAJ19943K2) overnight at 4°C. Fixed embryos were washed in 0.1% Tween 20 (Sigma, #P2287) in PBS and washed once with 1 mL 100% methanol (Fisher Scientific #A452) before being stored in 1 mL fresh 100% methanol at −20°C overnight. Stored larvae were rehydrated through a series of methanol dilutions before washing in 1 mL 1% Triton X-100 (Electron Microscopy Sciences #22140) in PBS (PBSTx). Larvae were permeabilized in 1 mL 50 μg/mL proteinase K (Fisher Scientific, #BP1700) in PBSTx for 30 min at room temperature. Permeabilized larvae were then refixed in 1 mL 4% PFA for 20 min at room temperature, washed in 1 mL PBSTx, and blocked with 1 mL blocking solution made with 10% normal goat serum (Cell Signaling 5425S) and 1% bovine serum albumin (Sigma, #A9647) in PBSTx for 5 h at room temperature. Larvae were then incubated with 1:50 anti-ZO1 monoclonal antibody (ZO1–1A12, ThermoFisher, #339100) overnight at room temperature. Larvae were washed in PBSTx, re-blocked in 1 mL 10% normal goat serum (Fisher Scientific, #NC9660079) in PBSTx for 1 h, and incubated in 1:400 goat anti-Mouse AF647 (Life Technologies, # A21237) in PBSTx overnight at room temperature. Larvae were washed again in 1 mL PBS before imaging.

#### Zebrafish larva microscopy and image analysis

For confocal imaging, larvae were embedded in 1.2% low melting-point agarose (IBI Scientific #IB70051).^72^ A series of z stack images with a 0.82–1 μm step size were generated through the brain using the Zeiss LSM 880 laser scanning microscope with an LD C-Apochromat 40× objective. Imaris (Bitplane Scientific Software) was used to measure fluorescence intensity and construct three-dimensional surface renderings. When comparing infected to uninfected vessels, threshold sizes and values were determined using the uninfected vessel and were then applied to the paired (usually contralateral) infected vessel in the same fish. When events were compared between larvae, identical confocal laser settings, software settings, and Imaris surface-rendering algorithms were used. For imaging blood vessels, transgenic animals with fluorescent blood vessels (Tg(*kdrl:GFP*),^30^ Tg(*fliE:GAL4;UAS:dsRed*),^76^ Tg(*fliE:GFP*),^76^ Tg(*flk:GFP*),^77^ Tg(*flt1:tomato*),^75^ and Tg(*flk:moesin-GFP*)^78^) were used, or Alexa 647 Dextran (ThermoFisher, #D22914) or FITC-Dextran (Invitrogen, #D1820) were injected intravenously. For imaging myeloid cells, transgenic animals with fluorescent myeloid cells (Tg(*mpeg1:dsRed*)^35^) were used.

For transmission electron microscopy, larvae were imaged by confocal microscopy in order to measure the distance from the top of the head to a region of interest containing a crossing microcolony. After larvae were rescued from 1.5% agarose used for confocal imaging, larvae were euthanized and fixed. Zebrafish larvae were incubated in a fixative solution (2% Paraformaldehyde + 2.5% Glutaraldehyde in 0.15 M Sodium cacodylate buffer pH 7.4) at room temperature for 30 min, then transferred to 4°C for 24 h. Samples were washed three times with 4°C 0.15 M Sodium cacodylate buffer pH 7.4 and post-fixed with 1% tetroxide osmium in 0.15 M Sodium cacodylate buffer pH 7.4 at 4°C. After 3 washes with cold double-distilled water, the fish were incubated in cold 2% uranyl acetate in double-distilled water for 2 h. Samples were then incubated in a series of 4°C solutions with increasing ethanol concentrations (50%, 70%, 90%, 3 times 100%) for 5 min each, then in room temperature 50% ethanol/50% acetone, and three times in 100% acetone. Next, the samples were infused with a mixture of 75% acetone/25% Durcupan (Sigma, #44610), then 50% acetone/50% Durcupan, and 25% acetone/75% Durcupan, for 2 h each. Then, the samples were incubated overnight at room temperature in 100% Durcupan and 3 times 2 h in 100% Durcupan. Finally, the samples were mounted with 100% Durcupan into an embedding mold, and oriented to be later sectioned along the frontal plane and cured at 60°C for 48 h. Sections were obtained using an ultramicrotome Leica UC6. To find the region of interest, every 10 μm, a 500 nm thick section was stained with toluidine blue and observed by light microscopy. Using the blood vessels as space markers and comparing their relative position in the 3D confocal volume, we decided when to start collecting 70 nm serial sections covering the area containing the bacterial microcolony.

Transmission electron micrographs were acquired using a JEOL 1400 plus operated at 80KeV and equipped with a Gatan One-view camera.

The quantity of bacilli per microcolony in vivo was determined by generating 3D renderings and acquiring the volumes of single bacilli and microcolonies with Imaris software. The volume of a microcolony was divided by the volume of a single bacillus to determine how many bacilli were within a microcolony.

### QUANTIFICATION AND STATISTICAL ANALYSIS

Most experiments were repeated multiple times to ensure reproducibility. The number of experimental replicates is indicated in the corresponding figure legend. If no number is listed, the experiment was conducted once. The following statistical analyses were performed using Prism 8 (GraphPad): Student’s and paired *t* test, Mann-Whitney U-test, and Fisher’s exact test. The statistical tests used for each figure can be found in the corresponding figure legend. The *n* values for larvae and microcolonies are given below each corresponding graph.

## Supplementary Material

1

2

3

Supplemental information can be found online at https://doi.org/10.1016/j.celrep.2025.116661.

## Figures and Tables

**KEY RESOURCES TABLE:** 

REAGENT or RESOURCE	SOURCE	IDENTIFIER

Antibodies		

anti-ZO1 Monoclonal (ZO1-1A12)	ThermoFisher	Cat# 339100; RRID: AB_2663169
Goat anti-Mouse AF647	Life Technologies	Cat# A21237

Bacterial and virus strains		

*Mycobacterium marinum* M strain transformed with pTEC18 or pTEC27	Takaki et al., 2013^72^	derivatives of ATCC #BAA-535
Δ*esx-1 M. marinum* M strain transformed with pTEC18 or pTEC27	Conrad et al., 2017^46^	N/A
*M. marinum* M strain *pknD*::Tn6042 transformed with pTEC18 or pTEC27	This paper	N/A
*M. marinum* transformed with pDL4912 (*msp12*:*dsRedII*;*map49*:*GFP*)	Cosma et al., 2004^38^	N/A
*M. tuberculosis* Δ*leuD*Δ*panCD* mc^2^ 6206 transformed with pTEC27	Roca et al., 2019^73^	N/A
*M. marinum* M strain *pcaA*::Tn20324 transformed with pTEC18 or pTEC27	This paper	N/A
Δ*erp M. marinum* M strain transformed with pTEC27	Takaki et al. 2013^72^	N/A
*M. marinum* M strain *pcaA*::Tn20324:*pcaA* - *pcaA*::*Tn20324* transformed with *pmsp12*:*cerulean*;*hsp60*:*pcaA*	Walton et al. 2018.^54^	N/A
*M. marinum* M strain *pknD*::Tn6042:*pknD* *pknD*::Tn6042 transformed with pJKS226	This paper	N/A
*M. tuberculosis* Δ*leuD*Δ*panCD* mc^2^ 6206 Δ*pknD* transformed with pTEC31	This paper	N/A
*M. tuberculosis* Δ*leuD*Δ*panCD* mc^2^ 6206 Δ*pcaA* transformed with pTEC31	This paper	N/A
*Mycobacterium smegmatis* mc^2^ 155 transformed with pTEC27	This paper	N/A
*M. smegmatis* mc^2^ 155 Δ*MSMEG_1351* transformed with pTEC27	This paper	N/A

Chemicals, peptides, and recombinant proteins		

Sodium chloride	JT Baker	Cat# 3628-F7
Potassium chloride	Sigma	Cat# P3911
Calcium chloride	G-Biosciences	Cat# RC-030
Magnesium sulfate heptahydrate	MP Biomedicals	Cat# 194833
Methylene blue chloride	Millipore Sigma	Cat# 284
1-phenyl-2-thiourea (PTU)	Sigma-Aldrich	Cat# 189235
Clodronate liposomes	Liposoma	Cat# C-005
Paraformaldehyde solution, 4% in PBS	Fisher scientific	Cat# AAJ19943K2
Tango buffer	ThermoFisher	Cat# BY5
Fluorospheres carboxylate-modified, 0.02μm crimson (625/645)	Invitrogen	Cat# F8782
Middlebrook Low Melt Point agarose	IBI Scientific	Cat# IB70051
Phenol Red	Sigma -Aldrich	Cat# P3532
Syncaine	Syndel	Cat# 886-86-2
Triton X-100	Electron Microscopy Sciences	Cat# 22140
Propidium Iodide (1.0 mg/mL Solution in Water)	Invitrogen	Cat# P3566
Tween 20	Sigma	Cat# P2287
Proteinase K	Fisher Scientific	Cat# BP1700
Methanol	Fisher Scientific	Cat# A452
Normal goat serum	Fisher Scientific	Cat# NC9660079
FITC-Dextran	Invitrogen	Cat# D1820
Hoechst	Invitrogen	Cat# H21486
Durcupan		N/A
7H10 Middlebrook Agar Base	HiMedia	Cat# M199
7H9 Middlebrook Broth Base	Sigma-Aldrich	Cat# M0178
Hygromycin B (in PBS 50 mg/mL)	ThermoFisher	Cat# 10687010
Kanamycin monosulfate	TCI	Cat# K0047
Tween-80	Sigma	Cat# P1754
Glycerol	ThermoFisher	Cat# Pl17904
Bovine serum albumin	Sigma	Cat# A9647
Glucose	Sigma	Cat# D9434-500g
NaOH	Sigma	Cat# 221465
Oleic acid	Sigma	Cat# O1008
Albumin	Sigma	Cat# A9647
Catalase	Sigma-Aldrich	Cat# C1345-1G
Dextrose	Sigma-Aldrich	Cat# D9434-500g
L-leucine	Milipore	Cat# 4330-100GM
Calcium pantothenate	Sigma-Aldrich	Cat# PHR1232
Anhydrotetracycline hydrochloride	Acros Organics	Cat# 13803-65-1
Glycine	Sigma-Aldrich	Cat# G8898-1KG
Isovaleronitrile, 98%	Sigma-Aldrich	Cat# 308528-5G

Experimental models: Organisms/strains		

Zebrafish: wildtype AB	University of Washington University of California, Los Angeles	ZFIN ID: ZDB-GENO-960809-7
Zebrafish: Tg(*kdrl:GFP*)	Choi et al., 2007^30^	ZFIN ID: ZDB-TGCONSTRCT-070529-1
Zebrafish: Tg(*flk:GAL4;UAS:Life-Act-GFP*)	Mizoguchi et al., 2016^74^	N/A
Zebrafish: Tg(*flt1:Tomato*)	Hogan et al., 2009^75^	ZFIN ID: ZDB-TGCONSTRCT-110504-1
Zebrafish: Tg(*fliE:GFP*)	Lawson and Weinstein, 2002^76^	ZFIN ID: ZDB-TGCONSTRCT-070117-94
Zebrafish: Tg(*flk:GFP*)	Liu et al., 2007^77^	ZFIN ID: ZDB-TGCONSTRCT-070529-1
Zebrafish: Tg(*fliE:Gal4; UAS:dsRed*)	Lawson and Weinstein, 2002^76^	N/A
Zebrafish: Tg(*flk:alpha-catenin-GFP*)	Wang et al., 2010^78^	N/A
Zebrafish: Tg(*mpeg1:dsRed*)	Ellett et al., 2011^35^	ZFIN ID: ZDB-FISH-150901-6828
Zebrafish: Tg(*flk:moesin-GFP*)	Wang et al., 2010^78^	N/A

Oligonucleotides		

*pu.1* morpholino component 1, sequence: CCTCCATTCTGTACGGATGCAGCAT	Clay et al., 2007^34^	N/A
*pu.1* morpholino component 2, sequence: GGTCTTTCTCCTTACCATGCTCTCC	Clay et al., 2007^34^	N/A
*pknD*::Tn *M. marinum* sequencing F primer sequence: TAGCGTGAATATGTAGGGTC	This paper	N/A
*pknD*::Tn *M. marinum* sequencing R primer sequence: ATCTACACCGAGCTCACCAA	This paper	N/A
*mincle* sequencing F primer sequence: GAATTTCTGCGCTAGCCTG	This paper	N/A
*mincle* sequencing R primer sequence: GCTATGCTCTTGAATAAGATGTGC	This paper	N/A
MSMEG_1531del ultramer: GGGTTCGATGCTGCCCCCCGGTTTCTCCGCCGAGA GGTGCACGTCAGTGACCAAGCAGCCCGGAAAGCTA CAACCAGGTTTGTCTGGTCAACCACCGCGGTCTC AGTGGTGTACGGTACAAACCATCGACGTCAACCA GTTCACCCTGGCGAAGTAGCGGCTCTTCAGAT TCGCTATGCGGGACCTGCCCCGACACGATA	This paper	N/A
JS285 (add lox66/71 to pKM464 F primer sequence): GGCTTGTCGACGACGGCGGTCTCCGTCGTCAGGA TCATTACCGTTCGTATAGCATACATTATACGAAGTT ATCTCGAGTCTAGAGCATGCACTAGT	This paper	N/A
JS286 (add lox66/71 to pKM464 R primer sequence): TACCGTTCGTATAATGTATGCTATACGAAGT TATAAGCTTATCGATGTCGACGTAGT	This paper	N/A
JS360 (amplify *pknD^Mtb^* downstream R primer sequence): GGCTACGGTCTCAACGAGATATCACGCCCTGTACG	This paper	N/A
JS358 (amplify vector for GGA F primer sequence): GGCTACGGTCTCTCAAAGGCGGTAATACGGTTATC	This paper	N/A
JS359 (amplify vector for GGA R primer sequence): GGCTACGGTCTCATCGTGATACGCCTATTTTTATAG	This paper	N/A
JS361 (amplify *pknD^Mtb^* downstream F primer sequence): GGCTACGGTCTCTCACTAGTTCTAGAGAACGACCG AGTGGTGAAAC	This paper	N/A
JS362 (amplify *hygR* for GGA F primer sequence): GGCTACGGTCTCTAGTGGATCCATAACTTCG	This paper	N/A
JS363 (amplify *hygR* for GGA R primer sequence): GGCTACGGTCTCATTCCGCAGGTAGGGTCGC TCGAGGGTACCGGCGCG	This paper	N/A
JS364 (amplify *pknD^Mtb^* upstream R primer sequence): GGCTACGGTCTCAGGAACGGCATCGCTCACC	This paper	N/A
JS365 (amplify *pknD^Mtb^* downstream F primer sequence): GGCTACGGTCTCTTTTGGATATCCACAGATCCAAGCCC	This paper	N/A
JS372 (*ΔpknD M. tuberculosis* sequencing R primer sequence): GGTGGTGTTTCGTCCGCTTAC	This paper	N/A
JS373 (*ΔpknD M. tuberculosis* sequencing F primer sequence): ATGGAGCAGTTCGTCTATGCCTAC	This paper	N/A
JS444 (amplify *pcaA^Mtb^* downstream R primer sequence): GGCTACGGTCTCAACGAGATATCGGTGCCTTCGAAGCATTC	This paper	N/A
JS445 (amplify *pcaA^Mtb^* downstream F primer sequence): GGCTACGGTCTCGCACTACCGACGTCGACCAGTTCACA	This paper	N/A
JS446 (amplify *pcaA^Mtb^* upstream R primer sequence): GGCTACGGTCTCCGGAATCCAAAATGCGGCGTGAGCTG	This paper	N/A
JS447 (amplify *pcaA^Mtb^* upstream F primer sequence): GGCTACGGTCTCTTTTGGATATCGACAAGATCGGTTACGACG	This paper	N/A
JS448 (MSMEG_1351 deletion sequencing F primer sequence): ATCTTCTCCAGCAACAACCA	This paper	N/A
JS449 (MSMEG_1351 deletion sequencing R primer sequence): ACCTTGGACAGCTTTTCGATG	This paper	N/A
JS465 (*ΔpcaA M. tuberculosis* R primer sequence): AGACCCCACGATGGCTTACAC	This paper	N/A
JS466 (*ΔpcaA M. tuberculosis* sequencing F primer sequence): GCACATCGCCAACAACCTTAT	This paper	N/A
JS485 (linearise pMV261 F primer sequence): CATTGCGAAGTGATTCCTCC	This paper	N/A
JS486 (linearise pMV261 R primer sequence): TAGCGTACGATCGACTGC	This paper	N/A
JS487 (amplify *pknD^Mm^* F primer sequence): TCCCCGATCCGGAGGAATCACTTCGCAA TGGTGAGCGAGACCGGGCCG	This paper	N/A
JS488 (amplify *pknD^Mm^* R primer sequence): ATTTGATGCCTGGCAGTCGATCGTACGCTA TCAGGATCCTTGGGCCAGTTTCAG	This paper	N/A
JS493 (linearise pTEC27 F primer sequence): CACTTTCTGGCTGGATGATG	This paper	N/A
JS494 (linearise pTEC27 R primer sequence): CCACGGTTGATGAGAGCT	This paper	N/A
JS495 (amplify *hsp60:pknD^Mm^* F primer sequence): CACCTACAACAAAGCTCTCATCAACCGTGGAAA TCTAGACGGTGACCACAACG	This paper	N/A
JS496 (amplify *hsp60:pknD^Mm^* R primer sequence): TGAATCGCCCCATCATCCAGCCAGAAAGT GTTGTTGGCTAGCTGATCACC	This paper	N/A

Recombinant DNA		

pTEC18 (*msp12:eBFP2*)	Takaki et al., 2013^72^	Addgene#30177
pTEC27 (*msp12:tdTomato;hygR*)	Takaki et al., 2013^72^	Addgene #30182
pTEC31 (*msp12:tdTomato;kanR*)	Conrad et al., 2017^46^	N/A
pNitET-SacB-Kan	Murphy et al., 2015^79^	Addgene#107692
pKM461	Murphy et al., 2018^80^	Addgene #108320
pCre-SacB-Zeo	Murphy et al., 2015^79^	Addgene#107706
pJKS146 (pKM464 [Addgene #108322] modified to have lox66/71 sites flanking attB)	This paper	N/A
pJKS181 (pKM342 [Addgene #71486] with inserts to delete *pknD*)	This paper	N/A
pJKS217 (pKM342 [Addgene #71486] with inserts to delete *pcaA*)	This paper	N/A
pJKS226 (*msp12:tdTomato;hsp60:pknD^Mm^*)	This paper	N/A

gRNAs		

Mincle gRNA 1: GGTGAAGAGAAGCGTAACTT	This paper	N/A
Mincle gRNA 2: GAGATCTGTGACACTCAAAT	This paper	N/A
Mincle gRNA 3: AGCCTAACATGGGTCTGCAG	This paper	N/A

Software and algorithms		

Imaris	Bitplane	https://imaris.oxinst.com/
Adobe Illustrator	Adobe	https://www.adobe.com/products/illustrator.html
Prism	GraphPad	https://www.graphpad.com/
ImageJ	ImageJ	https://imagej.net/
FPC (ImageJ); macro for quantification of bacterial burden by fluorescence imaging	Takaki et al., 2013^72^	N/A
Zen Black	Zeiss	N/A

## Highlights

- Macrophages release mycobacteria into the blood, where they bind to brain endothelial cells
- Mycobacteria attach to the endothelium using trehalose dimycolate (TDM)
- TDM binds its host receptor, Mincle, triggering cell junction opening
- Mycobacteria cross the blood-brain barrier using a paracellular mechanism
