# Supplementary material for: Mycobacteria trehalose dimycolate interactions with host Mincle remodel blood-brain barrier junctions for brain invasion
Source: Cell Rep. Author manuscript; Available in PMC 2026 Feb 2. (PMC12862661; doi:10.1016/j.celrep.2025.116661)
Supplement: 1 [file NIHMS2132753-supplement-1.pdf]

Cell Reports, Volume *44*

Supplemental information

Mycobacteria trehalose dimycolate interactions with host Mincle remodel blood-brain barrier junctions for brain invasion

Megan I. Hayes, Sumedha Ravishankar, Jonathan K. Shanahan, Adam J. Fountain, Lalita Ramakrishnan, and Cressida A. Madigan

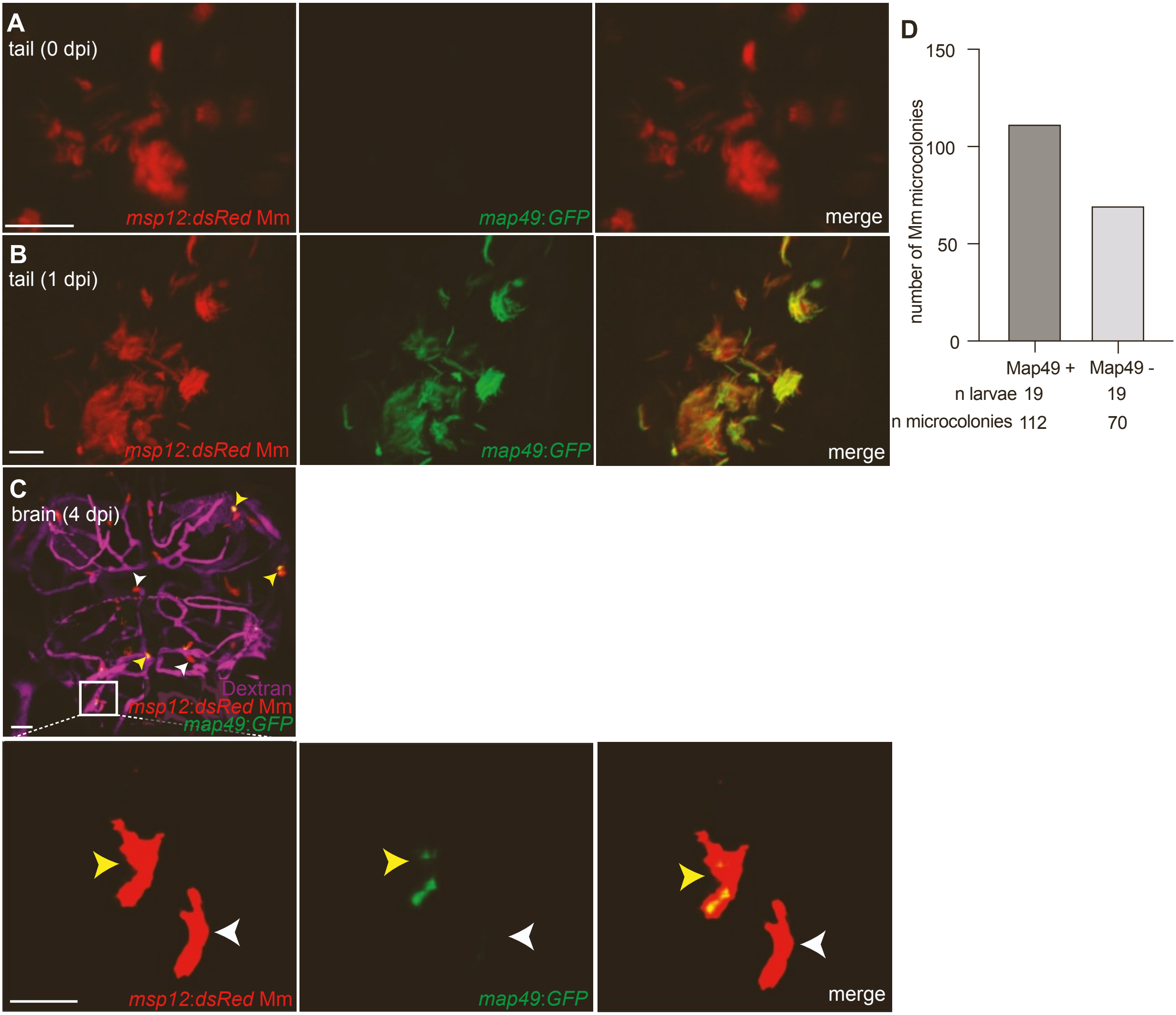
A
tail (0 dpi)
msp12:dsRed Mm
map49:GFP
merge
B
tail (1 dpi)
msp12:dsRed Mm
map49:GFP
merge
C
brain (4 dpi)
Dextran
msp12:dsRed Mm
map49:GFP
msp12:dsRed Mm
map49:GFP
merge
D
number of Mm microcolonies
150
100
50
0
Map49 +
Map49 -
n larvae 19
19
n microcolonies 112
70

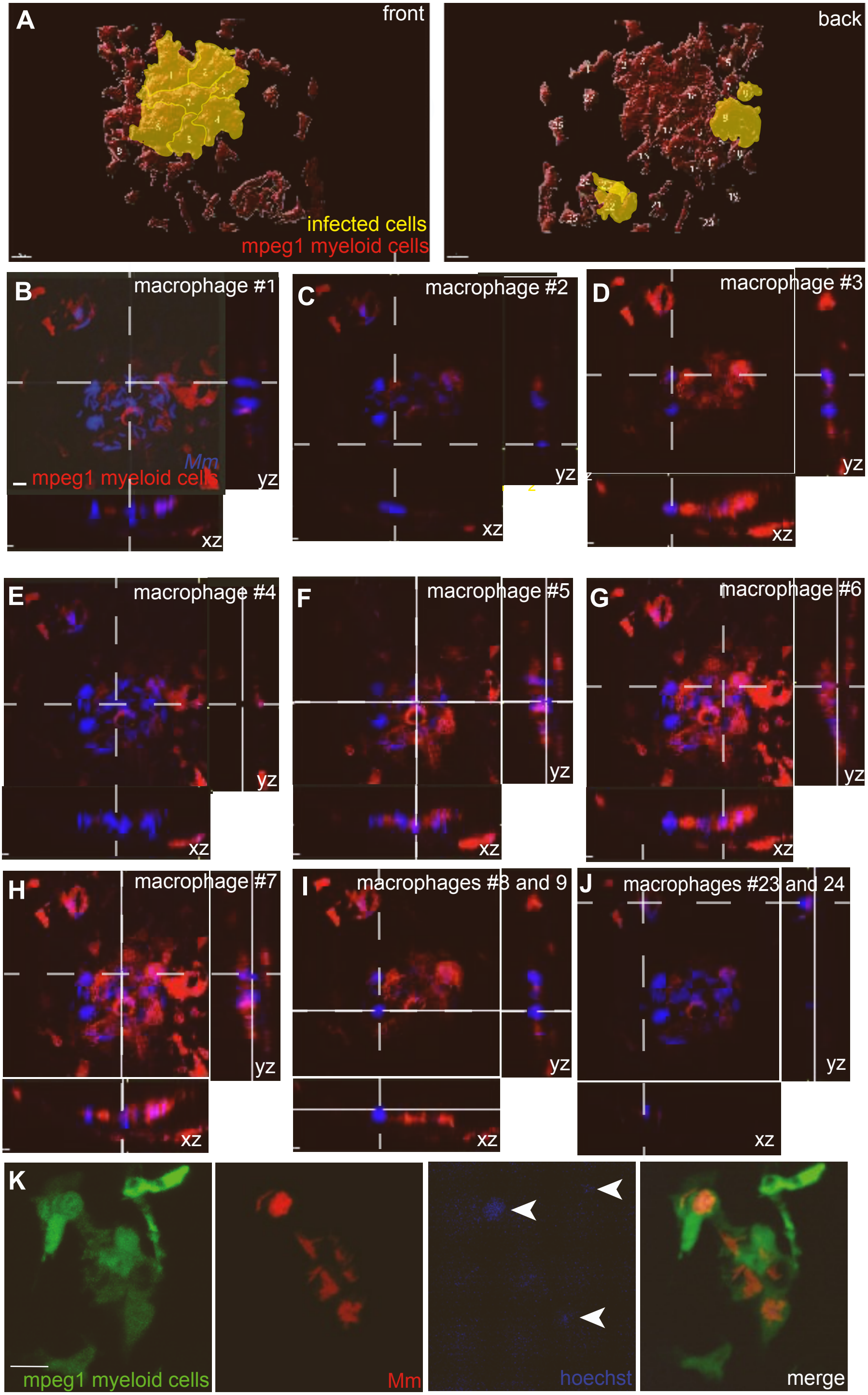
A
front
infected cells
mpeg1 myeloid cells
back
B
macrophage #1
Mm
mpeg1 myeloid cells
yz
xz
C
macrophage #2
yz
xz
D
macrophage #3
yz
xz
E
macrophage #4
yz
xz
F
macrophage #5
yz
xz
G
macrophage #6
yz
xz
H
macrophage #7
yz
xz
I
macrophages #8 and 9
yz
xz
J
macrophages #23 and 24
yz
xz
K
mpeg1 myeloid cells
Mm
hoechst
merge

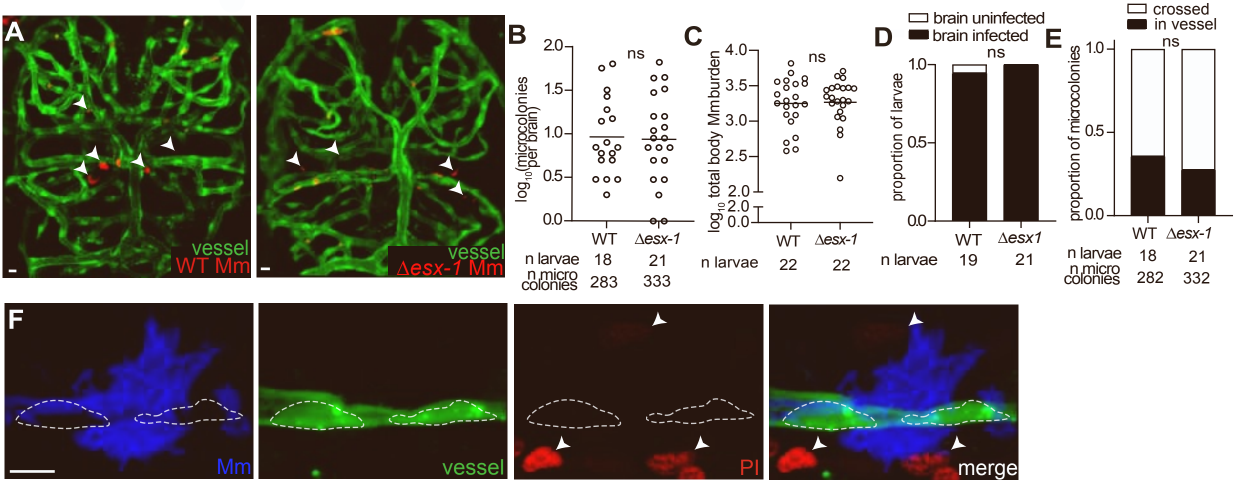
A
vessel
WT Mm
vessel
Δesx-1 Mm
B
ns
log10(microcolonies per brain)
2.0
1.5
1.0
0.5
0.0
WT
Δesx-1
n larvae 18 21
n micro colonies 283 333
C
ns
log10 total body Mm burden
4.0
3.5
3.0
2.5
2.0
2.0
0.0
WT
Δesx-1
n larvae 22 22
D
brain uninfected
brain infected
ns
proportion of larvae
1.0
0.5
0.0
WT
Δesx1
n larvae 19 21
E
crossed
in vessel
ns
proportion of microcolonies
1.0
0.5
0.0
WT
Δesx-1
n larvae 18 21
n micro colonies 282 332
F
Mm
vessel
PI
merge

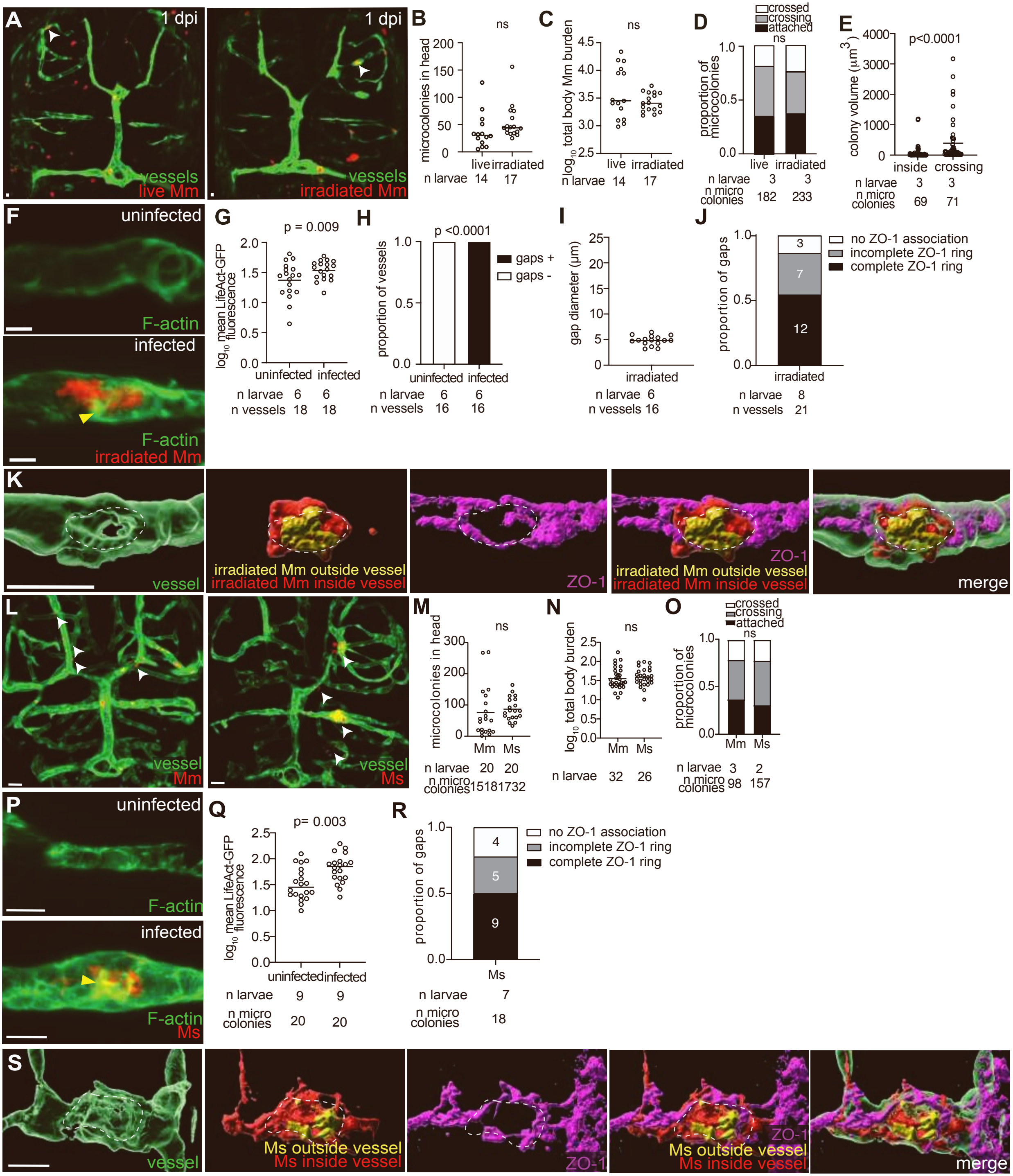

A
1 dpi
vessels
live Mm
1 dpi
vessels
irradiated Mm
B
ns
microcolonies in head
live irradiated
n larvae 14 17
C
ns
log10 total body Mm burden
live irradiated
n larvae 14 17
D
crossed
crossing
attached
ns
proportion of microcolonies
live irradiated
n larvae 3 3
n micro colonies 182 233
E
p<0.0001
colony volume (μm3)
inside crossing
n larvae 3 3
n micro colonies 69 71
F
uninfected
F-actin
infected
F-actin
irradiated Mm
G
p = 0.009
log10 mean LifeAct-GFP fluorescence
uninfected infected
n larvae 6 6
n vessels 18 18
H
p <0.0001
proportion of vessels
gaps +
gaps -
uninfected infected
n larvae 6 6
n vessels 16 16
I
gap diameter (μm)
irradiated
n larvae 6
n vessels 16
J
proportion of gaps
no ZO-1 association
incomplete ZO-1 ring
complete ZO-1 ring
3
7
12
irradiated
n larvae 8
n vessels 21
K
vessel
irradiated Mm outside vessel
irradiated Mm inside vessel
ZO-1
ZO-1
irradiated Mm outside vessel
irradiated Mm inside vessel
merge
L
vessel
Mm
vessel
Ms
M
ns
microcolonies in head
Mm Ms
n larvae 20 20
n micro colonies 1518 1732
N
ns
log10 total body burden
Mm Ms
n larvae 32 26
O
crossed
crossing
attached
ns
proportion of microcolonies
Mm Ms
n larvae 3 2
n micro colonies 98 157
P
uninfected
F-actin
infected
F-actin
Ms
Q
p= 0.003
log10 mean LifeAct-GFP fluorescence
uninfected infected
n larvae 9 9
n micro colonies 20 20
R
proportion of gaps
no ZO-1 association
incomplete ZO-1 ring
complete ZO-1 ring
4
5
9
Ms
n larvae 7
n micro colonies 18
S
vessel
Ms outside vessel
Ms inside vessel
ZO-1
ZO-1
Ms outside vessel
Ms inside vessel
merge

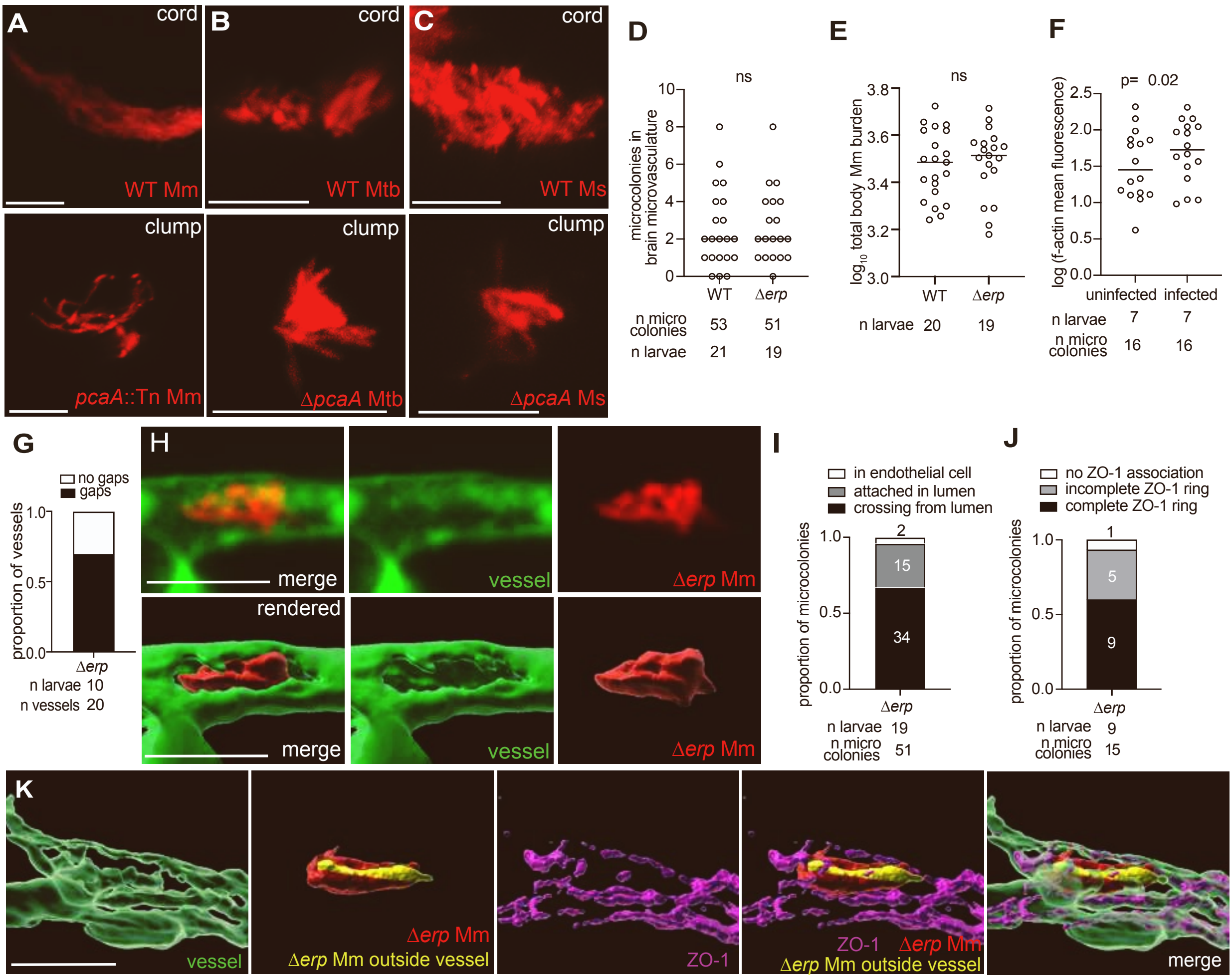
A
cord
WT Mm
B
cord
WT Mtb
C
cord
WT Ms
clump
pcaA::Tn Mm
clump
ΔpcaA Mtb
clump
ΔpcaA Ms
D
ns
microcolonies in brain microvasculature
WT
Δerp
n micro colonies 53 51
n larvae 21 19
E
ns
log10 total body Mm burden
WT
Δerp
n larvae 20 19
F
p= 0.02
log (f-actin mean fluorescence)
uninfected
infected
n larvae 7 7
n micro colonies 16 16
G
no gaps
gaps
proportion of vessels
Δerp
n larvae 10
n vessels 20
H
merge
vessel
Δerp Mm
rendered
merge
vessel
Δerp Mm
I
in endothelial cell
attached in lumen
crossing from lumen
proportion of microcolonies
2
15
34
Δerp
n larvae 19
n micro colonies 51
J
no ZO-1 association
incomplete ZO-1 ring
complete ZO-1 ring
proportion of microcolonies
1
5
9
Δerp
n larvae 9
n micro colonies 15
K
vessel
Δerp Mm
Δerp Mm outside vessel
ZO-1
ZO-1 Δerp Mm
Δerp Mm outside vessel
merge

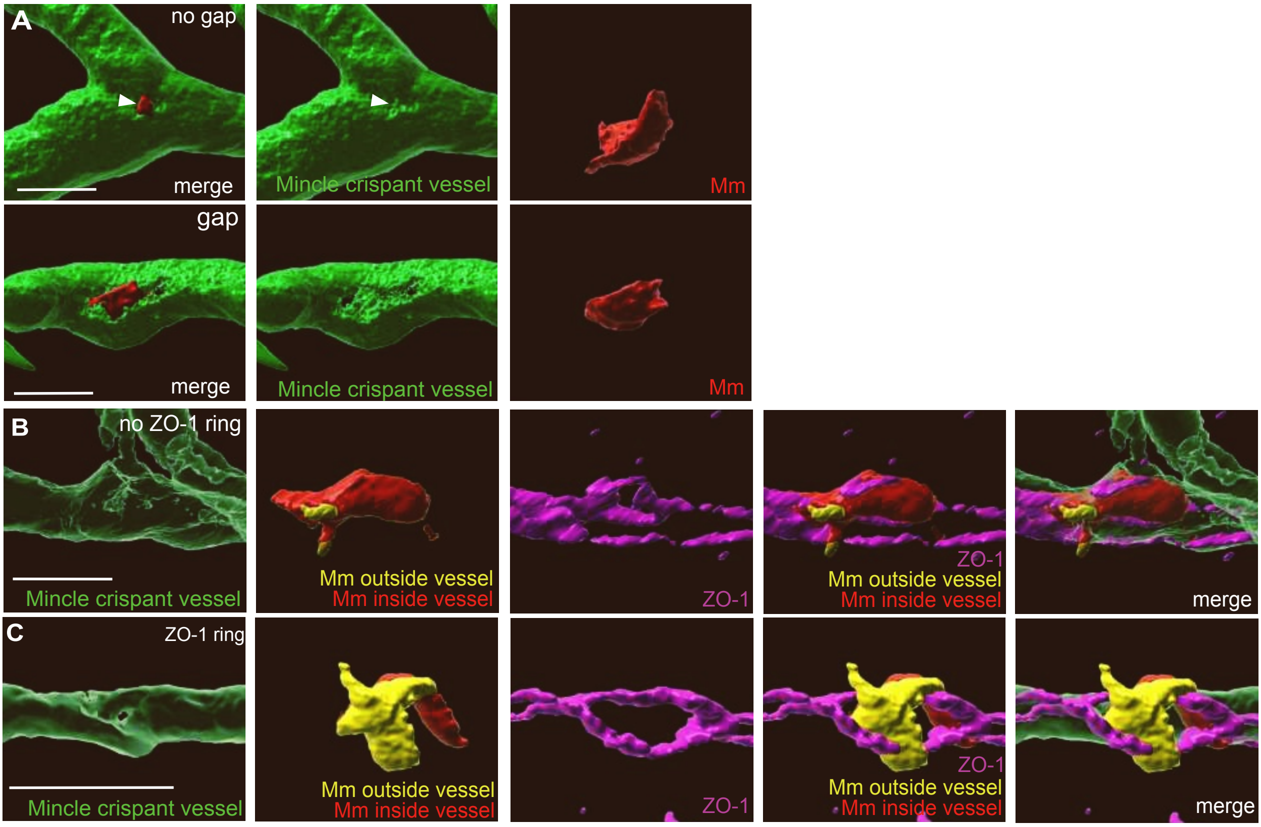
A
no gap
merge
Mincle crispant vessel
Mm
gap
merge
Mincle crispant vessel
Mm
B
no ZO-1 ring
Mincle crispant vessel
Mm outside vessel
Mm inside vessel
ZO-1
ZO-1
Mm outside vessel
Mm inside vessel
merge
C
ZO-1 ring
Mincle crispant vessel
Mm outside vessel
Mm inside vessel
ZO-1
ZO-1
Mm outside vessel
Mm inside vessel
merge

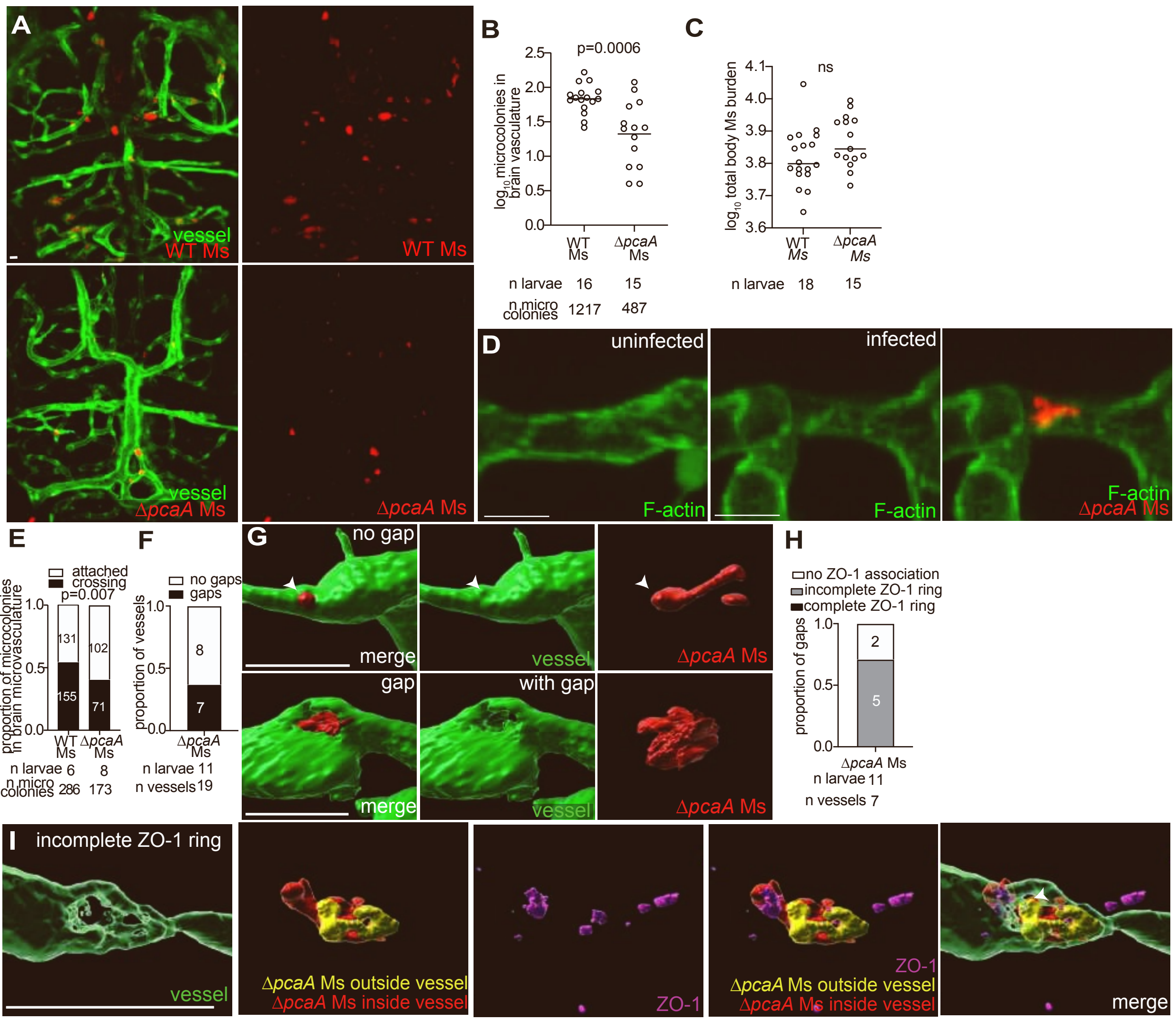
A
vessel
WT Ms
WT Ms
vessel
ΔpcaA Ms
ΔpcaA Ms
B
p=0.0006
log10 microcolonies in brain vasculature
2.5
2.0
1.5
1.0
0.5
0.0
WT Ms
ΔpcaA Ms
n larvae 16 15
n micro colonies 1217 487
C
ns
log10 total body Ms burden
4.1
4.0
3.9
3.8
3.7
3.6
WT Ms
ΔpcaA Ms
n larvae 18 15
D
uninfected
infected
F-actin
F-actin
F-actin
ΔpcaA Ms
E
attached
crossing
p=0.007
proportion of microcolonies in brain microvasculature
1.0
0.5
0.0
131
155
102
71
WT Ms
ΔpcaA Ms
n larvae 6 8
n micro colonies 286 173
F
no gaps
gaps
proportion of vessels
1.0
0.5
0.0
8
7
ΔpcaA Ms
n larvae 11
n vessels 19
G
no gap
merge
vessel
ΔpcaA Ms
gap
merge
with gap
vessel
ΔpcaA Ms
H
no ZO-1 association
incomplete ZO-1 ring
complete ZO-1 ring
proportion of gaps
1.0
0.5
0.0
2
5
ΔpcaA Ms
n larvae 11
n vessels 7
I
incomplete ZO-1 ring
vessel
ΔpcaA Ms outside vessel
ΔpcaA Ms inside vessel
ZO-1
ZO-1
ΔpcaA Ms outside vessel
ΔpcaA Ms inside vessel
merge

Supplemental Figures

Figure S1. Extracellular mycobacteria invading the brain derive from formerly intracellular mycobacteria.

(**A-B**) Representative confocal images of larvae tails infected with ~100 CFU Mm-*msp12*:*dsRed*;*map49*:*GFP* at 0 dpi (A) and 1 dpi (B). Red-fluorescent Mm (Map-), not previously internalized by monocytes. Red and green-fluorescent Mm (Map49+), previously or currently internalized by monocytes. Scale bar, 10µm. (**C**) Representative confocal image of brain vasculature (magenta fluorescent) at 4 dpi from larva in (A). White arrowhead, Map49-; Yellow arrowheads, Map49+. Boxed area is magnified in bottom panel. Scale bars, 10µm. (**D**) Number of Map49+ and Map49- Mm microcolonies from (C) in the brain. Representative of 2 independent experiments.

Figure S2. Rich foci contain infected and uninfected cells.

(**A**) Front (left) and back (right) 3D render of the granuloma in Figure 2F from 3 dpi larva with red-fluorescent myeloid cells. Yellow rendering, myeloid cells infected with Mm. Red rendering, uninfected myeloid cells. Scale bar, 10µm. (**B-J**) Representative confocal images with optical cross sections (yz, xz) of red-fluorescent myeloid cells infected with blue-fluorescent Mm from granuloma in (A). Scale bar, 10 µm. **(K)** Representative confocal images of green-fluorescent myeloid cells infected with red Mm from larvae intravenously injected with Hoechst. Arrowheads indicate Hoechst+ myeloid cells. Scare bar, 10 µm.

Figure S3. ***M. marinum* cross brain blood vessel independent of ESX-1 and do not lyse endothelial cells during brain invasion.**

(A) Representative confocal images of green-fluorescent brain vasculature in 2 dpi larva infected with ~1300 CFU red-fluorescent wildtype (WT) Mm (left) or ~800 CFU red-fluorescent Δ*esx-1* Mm (right). Arrowheads, microcolonies that have exited the vasculature and entered the brain. Scale bar, 10 µm. **(B)** Total WT Mm and Δ*esx-1* Mm microcolonies per larva head from experiment in (A). Horizontal bars, means; ns: not significant. Student's t-test. **(C)** Mm burden per larva from (A) at 2 dpi quantified by fluorescent pixel counts (FPC). Horizontal bars, means; ns: not significant, Student's t-test. **(D)** Proportion of larva from (A) with (black) or without (white) WT Mm or Δ*esx-1* Mm microcolonies in the brain; ns: not significant, Fisher's exact test. **(E)** Proportion of WT Mm and Δ*esx-1* Mm microcolonies in blood vessels (black) or in the brain (white) from larvae in (A); ns: not significant. Fisher's exact test. (**F**) Representative confocal image of 3 dpi larva with green-fluorescent blood vessels infected with blue-fluorescent WT Mm and intravenously injected with propidium iodide (PI). Dashed circles indicate nuclei of vascular endothelial cells. Arrowheads, PI stained cells in brain. Scale bar, 10µm. All data representative of 2 independent experiments.

Figure S4. Pathogenic mycobacteria cross the blood-brain barrier using cell surface determinants shared with nonpathogenic mycobacteria

(**A)** Representative confocal images of green-fluorescent brain vasculature in 1 dpi larvae infected with live (left) or γ-irradiated (right) red-fluorescent Mm. Arrowheads, representative Mm microcolonies. Scale bar, 10µm. **(B)** Total live or γ-irradiated Mm microcolonies per larva head at 1 dpi from experiment in (A). Horizontal bars, means; ns: not significant, Student's t-test. Representative of 2 independent experiments. **(C)** Live or γ-irradiated burden per larva at 1 dpi quantified by fluorescent pixel counts (FPC) from (A). Horizontal bars, means; ns: not significant,

Student's t-test. Representative of 2 independent experiments. **(D)** Proportion of live and γ-irradiated Mm microcolonies that are attached (black), crossing (gray), and crossed (white) into the brain; ns: not significant, Fisher's exact test. Representative of 2 independent experiments. **(E)** Volume of attached and crossing red-fluorescent γ-irradiated Mm microcolonies at 1 dpi. Horizontal bars, means, Student's t-test. **(F)** Representative confocal images of uninfected (top) and red-fluorescent γ-irradiated Mm infected (bottom) vessels from *flk:GAL4;UAS:LifeAct-GFP* larvae at 1 dpi. Arrowhead, green-fluorescent F-actin accumulation around microcolony. Scale bar, 10μm. **(G)** Quantification of F-actin in γ-irradiated Mm infected vessels compared to contralateral uninfected vessels from the same animal at 1 dpi. Horizontal bars, means; paired t-test. **(H)** Proportion of vessels associated with blood vessel gaps in uninfected and γ-irradiated Mm infected vessels; Fisher's exact test. Representative of 2 independent experiments. **(I)** Maximum diameter of vessel gaps formed underneath γ-irradiated Mm microcolonies. Horizontal bar, mean. Representative of 2 independent experiments. **(J)** Proportion of gaps associated with a complete (black), incomplete (gray), or no ZO-1 ring (white) for γ-irradiated Mm microcolonies that are crossing blood vessels. **(K)** 3D rendered, representative confocal image from 1 dpi larva with green-fluorescent vessels infected with red-fluorescent γ-irradiated Mm, fixed and stained with anti-ZO-1 antibody (pseudo-colored magenta). Dashed circle, gap ringed by ZO-1 under γ-irradiated Mm microcolony. Yellow indicates parts of the microcolony that have exited the vasculature and entered the brain. Scale bar, 10μm. **(L)** Representative confocal images of *fliE:GFP* brain vasculature in 1 dpi larvae infected with red-fluorescent Mm (left) and *M. smegmatis* (Ms) (right). Arrowheads, representative microcolonies. Scale bar, 10 μm. **(M)** Total Mm or Ms microcolonies per larva head at 1 dpi. Horizontal bars, means; ns: not significant; Mann-Whitney U-test. **(N)** Mm or Ms burden per larva at 1 dpi quantified by FPC from experiment in

(L). Horizontal bars, means; ns: not significant, Student's t-test. **(O)** Proportion of Mm and Ms microcolonies that are attached (black), crossing (gray), and crossed (white) into the brain; ns: not significant, Fisher's exact test. **(P)** Representative confocal images of uninfected **(**top) and red-fluorescent Ms infected (bottom) vessels from *flk:GAL4;UAS:LifeAct-GFP* larvae at 1 dpi. Arrowhead, green-fluorescent F-actin accumulation around microcolony. Scale bar, 10µm. **(Q)** Quantification of F-actin in Ms infected vessels compared to contralateral uninfected vessels from the same animal at 1 dpi. Horizontal bars, means; paired t-test. **(R)** Proportion of gaps associated with a complete (black), incomplete (gray), or no ZO-1 ring (white) for Ms microcolonies that are crossing blood vessels. **(S)** 3D rendered, representative confocal image from 1 dpi larva with green-fluorescent vessels infected with red-fluorescent Ms, fixed and stained with anti-ZO-1 antibody (pseudo-colored magenta). Dashed circle, gap ringed by ZO-1 under Ms microcolony. Yellow indicates parts of the microcolony that have exited the vasculature and entered the brain. Scale bar, 10µm.

Figure S5. Δ*erp M. marinum* behaves like wildtype *M. marinum*.

(A) Representative confocal images of red-fluorescent WT Mm (top) and red-fluorescent *pcaA*::Tn Mm (bottom) microcolonies in larva brains. Microcolonies show a cord (WT) or clump (*pcaA*::Tn) morphology. Scale bar, 10µm. **(B)** Representative confocal images of red-fluorescent WT *M. tuberculosis* (Mtb, top) and red-fluorescent Δ*pcaA* Mtb (left) microcolonies in larva brains. Microcolonies show a cord (WT) or clump (Δ*pcaA*) morphology. Scale bar, 10µm. **(C)** Representative confocal images of red-fluorescent WT *M. smegmatis* (Ms) (top) and red-fluorescent Δ*pcaA* Ms (bottom) microcolonies in larva brains. Microcolonies show a cord (WT) or clump (*pcaA*::Tn) morphology. Scale bar, 10µm. **(D)** Total WT and Δ*erp* Mm microcolonies in

brain vasculature (attached and crossing microcolonies). Horizontal bars, means; ns: not significant; Student's t-test. **(E)** WT or Δ*erp* Mm burden per larva at 2 dpi quantified by fluorescent pixel counts (FPC). Horizontal bars, means; ns: not significant, Student's t-test. **(F)** Quantification of F-actin in Δ*erp* Mm infected vessel compared to contralateral uninfected vessel from the same animal. Horizontal bars, means; paired t-test. **(G)** Proportion of vessels associated with blood vessel gaps in Δ*erp* Mm infected vessels. Representative of 2 independent experiments. **(H)** Representative confocal image (top) and 3D rendering (bottom) of larvae with green-fluorescent blood vessels infected with red-fluorescent Δ*erp* Mm. Bottom, *pcaA*::Tn Mm microcolony crossing through a gap. **(I)** Proportion of vessels containing *pcaA*::Tn Mm that are attached (gray), crossing (black), or inside of an endothelial cell (white). **(J)** Proportion of gaps associated with a complete (black), incomplete (gray), or no ZO-1 ring (white) for Δ*erp* Mm microcolonies that are crossing blood vessels. **(K)** 3D rendered, representative confocal images from 3 dpi larva with green-fluorescent vessels infected with red-fluorescent Δ*erp* Mm*,* fixed and stained with anti-ZO-1 antibody (pseudo-colored magenta). Δ*erp* Mm microcolony with associated gap, ringed by ZO-1. Yellow indicates parts of the microcolony that have exited the vasculature and entered the brain. Scale bar, 10μm.

Figure S6. Host Mincle mediates junctional remodeling

(A) 3D rendered, representative confocal image of 2 dpi Mincle crispant larvae with green-fluorescent blood vessels infected with red-fluorescent Mm. Top, Mm microcolony in Mincle crispant larva crossing blood vessel without apparent gap (arrowheads). Bottom, Mm microcolony in Mincle crispant larva crossing through a gap. Scale bar, 10μm. **(B-C)** 3D rendered, representative confocal images from 2 dpi Mincle crispant larva with green-fluorescent vessels

infected with red-fluorescent Mm, fixed and stained with anti-ZO-1 antibody (pseudo-colored magenta). Mm microcolony without associated gap or ZO-1 ring (B). Mm microcolony with associated gap, partially ringed by ZO-1 (C). Yellow indicates parts of the microcolony that have exited the vasculature and entered the brain. Scale bar, 10μm.

Figure S7. Δ*pcaA M. smegmatis* behaves like *pcaA*::Tn *M. marinum*

(A) Representative confocal images of green-fluorescent brain vasculature in 1 dpi larvae infected with ~1000 CFU WT (top) or ~1,500 CFU Δ*pcaA* (bottom) red-fluorescent *M. smegmatis* (Ms). Scale bar, 10μm. **(B)** Total WT and Δ*pcaA* Ms microcolonies in brain vasculature (attached and crossing microcolonies). Horizontal bars, means; Student's t-test. **(C)** WT or Δ*pcaA* Ms burden per larva at 1 dpi quantified by fluorescent pixel counts (FPC) from experiment in (A). Horizontal bars, means; ns: not significant, Student's t-test. **(D)** Representative confocal images of red-fluorescent Δ*pcaA* Ms infected vessels from *flk:GAL4;UAS:LifeAct-GFP* larvae at 1 dpi. Scale bar, 10μm. **(E)** Proportion of attached or crossing WT and Δ*pcaA* Ms microcolonies (excluding microcolonies in the brain) in blood vessels from larva in (A); Fisher's exact test. **(F)** Proportion of vessels associated with gaps in Δ*pcaA* Ms infected vessels. **(G)** 3D rendered, representative confocal image of 3 dpi larvae with green-fluorescent blood vessels infected with red-fluorescent Δ*pcaA* Ms. Top, Δ*pcaA* Ms microcolony crossing blood vessel without apparent gap (arrowheads). Bottom, Δ*pcaA* Ms microcolony crossing through a gap. Scale bar, 10μm. **(H)** Proportion of gaps associated with a complete (black), incomplete (gray), or no ZO-1 ring (white) for Δ*pcaA* Ms microcolonies that are crossing blood vessels. **(I)** 3D rendered, representative confocal images from 2 dpi larva with green-fluorescent vessels infected with red-fluorescent Δ*pcaA* Ms, fixed and stained with anti-ZO-1 antibody (pseudo-colored magenta). Δ*pcaA* Ms microcolony with

associated gap, partially ringed by ZO-1. Yellow indicates parts of the microcolony that have exited the vasculature and entered the brain. Scale bar, 10μm.
